# Device engineering for photocurrent detected magnetic resonance and scanning probes using solid-state spin defects

**DOI:** 10.1080/14686996.2026.2691682

**Published:** 2026-08-17

**Authors:** Hiroki Morishita, Naoya Morioka, Eikichi Kimura, Keigo Arai, Yuichi Yamazaki, Toshu An, Shigemi Mizukami, Norikazu Mizuochi

**Affiliations:** a Center for Science and Innovation in Spintronics, Tohoku University, Sendai, Miyagi, Japan; b WPI Advanced Institute for Materials Research, Tohoku University, Sendai, Miyagi, Japan; c Institute for Chemical Research, Kyoto University, Gokasho, Uji, Kyoto, Japan; d Center for Spintronics Research Network, Institute for Chemical Research, Kyoto University, Gokasho, Uji, Kyoto, Japan; e School of Engineering, Institute of Science Tokyo, Yokohama, Kanagawa, Japan; f National Institutes for Quantum Science and Technology, Takasaki, Gunma, Japan; g School of Materials Science, Japan Advanced Institute of Science and Technology, Nomi, Ishikawa, Japan

**Keywords:** Solid-state quantum sensor, quantum spin, diamond, silicon carbide, photoelectrical detection, quantum scanning probe

## Abstract

Solid-state spin defects provide a versatile platform for quantum sensing with nanoscale spatial resolution and room-temperature operation. Spin defects in diamond have enabled mature scanning-probe devices, while related defects in silicon carbide and hexagonal boron nitride are being actively explored for scalable sensing platforms. However, the sensitivity of practical and scanning-probe devices remains below that of optimized bulk systems. Although optical fluorescence detection is widely used, practical performance is often constrained by limitations in signal acquisition and readout efficiency, motivating continued efforts to improve readout technologies. This review surveys material platforms and optical and photoelectrical readout technologies for solid-state spin defects. We compare fluorescence- and photoelectric-based detection schemes in terms of readout fidelity, sensitivity, and scalability, and discuss how materials properties and carrier transport influence practical performance. These perspectives provide guidelines for improving readout efficiency and advancing high-sensitivity quantum sensors and scanning probes.

## IMPACT STATEMENT

This review examines photoelectrical readout of solid-state spin defects, addressing the materials and practical challenges underlying high-sensitivity quantum sensors and scanning probes.

## GRAPHICAL ABSTRACT

**Figure:**
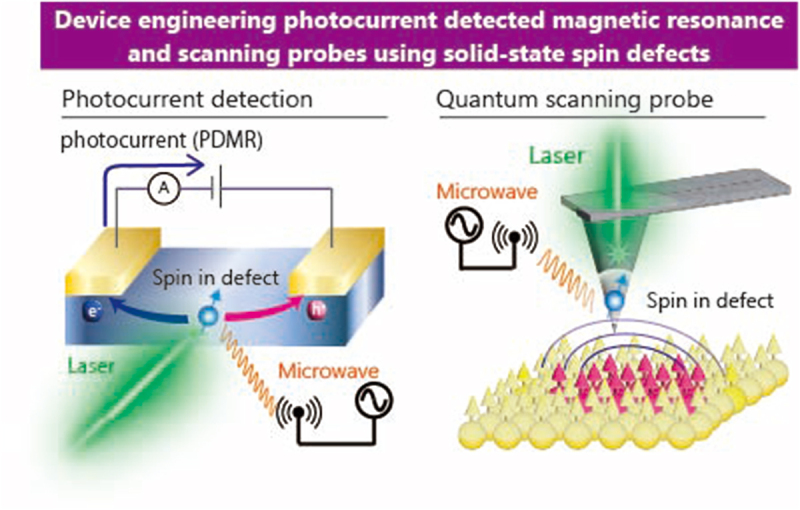

## 1. Introduction

Quantum sensing has emerged as a powerful approach for detecting extremely weak physical signals by exploiting quantum mechanical resources such as the quantum coherence of spin states [1]. Owing to their high sensitivity and non-invasive operation, quantum sensors enable measurements that are difficult or impossible with conventional classical probes, particularly when nanoscale spatial resolution and room-temperature functionality are simultaneously required. Among the various solid-state implementations, spin defects in wide-bandgap materials are especially attractive because of their robustness, scalability, and reliable operation under ambient conditions. Here, scalability mainly refers to ensemble scaling, namely the ability to increase the number of sensing defects or sensing elements contributing to the detected signal through materials growth and device fabrication. In this review, scalability refers to the possibility of increasing the number of sensing defects or sensing elements contributing to the detected signal through materials growth and device fabrication. Their atomic-scale dimensions enable highly localized measurements with nanometer spatial resolution, making them well suited for probing microscopic and spatially heterogeneous phenomena in condensed-matter systems. Nitrogen-vacancy (NV) centers in diamond represent one of the most mature and versatile platforms [1–3]. The NV electron spin can be optically initialized and read out while maintaining long coherence times even at room temperature, enabling sensitive detection of magnetic fields, electric fields, temperature, and strain [4]. When incorporated into thin membranes or nanostructures, NV centers further function as scanning quantum probes that bring the sensor into close proximity with a target sample [5–7]. Such platforms enable nanoscale magnetometry and current-density imaging with unprecedented spatial resolution [7–10]. Despite these advances and the recent commercialization of NV-based probes, the sensitivity of practical scanning implementations remains significantly below the limits demonstrated in optimized bulk materials. Commercial devices typically operate at sensitivities on the order of μT/$\sqrt{\mathrm{Hz}}$, whereas optimized sensors range from the nT/$\sqrt{\mathrm{Hz}}$ regime for single-spin devices down to the fT/$\sqrt{\mathrm{Hz}}$ regime for bulk ensemble systems under optimized conditions [11–13]. This performance gap persists even though both bulk and scanning implementations rely on optical fluorescence readout. In practice, device performance is influenced by several factors, including system-level constraints in miniaturized geometries and limitations in signal acquisition. Although fluorescence-based readout remains widely used and versatile, practical optical losses, background signals, and experimental overheads can reduce signal-to-noise ratios and scalability. Consequently, improving readout efficiency has become an important focus for enhancing the sensitivity of practical quantum sensors.

Beyond diamond, alternative host materials are being explored to address scalability and device-integration challenges. Silicon carbide (SiC) provides a particularly attractive semiconductor platform compatible with wafer-scale growth and established microfabrication processes, enabling large-area devices and straightforward electrical integration. In contrast, hexagonal boron nitride (hBN) offers an emerging two-dimensional host that allows atomically thin sensors and extreme sensor-sample proximity, opening opportunities for surface-sensitive measurements. However, compared with diamond and SiC, hBN-based spin defects remain at an earlier stage of technological maturity, particularly with respect to reproducible defect engineering and electrical readout implementations. Because this review emphasizes readout engineering for quantum sensors and scanning applications, we focus on the more established diamond and SiC platforms while discussing hBN as a promising future direction. To address the limitations of fluorescence detection, increasing attention has been directed to alternative readout mechanisms that convert spin information into electrical signals. One such approach is photoelectric detection of magnetic resonance (PDMR), in which spin-dependent photoionization processes generate a measurable photocurrent [14]. In this technique, optical excitation is used to drive the photoionization process, while the resulting signal is detected electrically. Thus, PDMR combines optical excitation with electrical readout. This mechanism is distinct from electrically detected magnetic resonance (EDMR), where spin-dependent transport or recombination processes are monitored electrically [15]. PDMR-based photocurrent detection can be implemented using on-chip electrodes and compact electronics, enabling efficient signal collection, reduced optical complexity, and potential compatibility with integrated and scanning-probe architectures, depending on device implementation. Such approaches provide a pathway toward scalable, chip-level quantum sensors with improved signal acquisition and system robustness.

Here we distinguish ODMR, EDMR, and PDMR to avoid confusion among related spin readout techniques. A representative comparison relevant to spin-defect quantum sensors is summarized in Table 1. ODMR detects magnetic resonance through spin-dependent optical observables, such as fluorescence, absorption, or polarization signals [4,16,17]. EDMR is used here in a broader sense to denote resonance detection through spin-dependent electrical observables, including current [15,18–22], photocurrent [23–26], capacitance [27], and related responses [28]. PDMR refers to a photoelectric readout scheme in which optical excitation drives spin-dependent photoionization; the resulting signal is detected electrically, typically as a photocurrent [14]. Although these approaches may share experimental components such as optical excitation or microwave driving depending on the material platform, they are distinguished here by their dominant readout mechanisms. In this review, we primarily focus on fluorescence-based ODMR and PDMR as representative readout schemes for solid-state spin-defect quantum sensors, while EDMR is included mainly to clarify the broader landscape of electrical spin readout techniques.

**Table 1.** Comparison of detection methods of ODMR, EDMR, and PDMR for quantum spin in solid.

Method	Spin-dependent process	Typical excitation	Key features	Practical constraints
ODMR	Optical contrast	Optical + MW	Optical observables (e.g. photoluminescence, absorption, polarization)	Optical access and signal collection required
EDMR	Electrical responses	Electrical and/or optical + MW	Electrical observables (e.g. current, photocurrent, capacitance, noise)	Observable and implementation depend on material platform and device structure
PDMR	Photoionization	Optical + MW	Photocurrent-based electrical readout	Requires optical excitation and charge collection structures

This review surveys material platforms and readout technologies for solid-state spin defects, with an emphasis on engineering optical and photoelectrical signal acquisition for compact, scanning-compatible devices. It is organized as follows. Section 2 introduces the material platforms, including NV centers in diamond, silicon vacancy (V${\;}_{\mathrm{Si}}$) defects in SiC, and boron vacancy (V${\;}_{B}$) centers in hBN. Section 3 describes the operating principles of spin-defect sensors, including spin initialization, manipulation, optical readout, and sensing performance. Section 4 discusses photoelectrical detection techniques, carrier transport, contact engineering, and charge-state stability. Section 5 presents scanning-probe fabrication methods and associated limitations. Section 6 compares optical and photoelectrical readout schemes in terms of readout fidelity, sensitivity, scalability, and practical constraints. Section 7 summarizes representative demonstrations in diamond and SiC. Finally, Section 8 concludes the review and outlines future directions.

## 2. Material platforms of spin-defect quantum sensors

Quantum sensors are generally classified into three types according to Degen et al. [1]:

1. Sensors that employ quantum objects or quantum states,
2. Sensors that exploit quantum coherence,
3. Sensors that utilize quantum entanglement.

While type-3 sensors may ultimately surpass classical sensitivity limits, this review focuses on type-1 and type-2 sensors, which are more practical with current solid-state systems.

**Figure 1.**
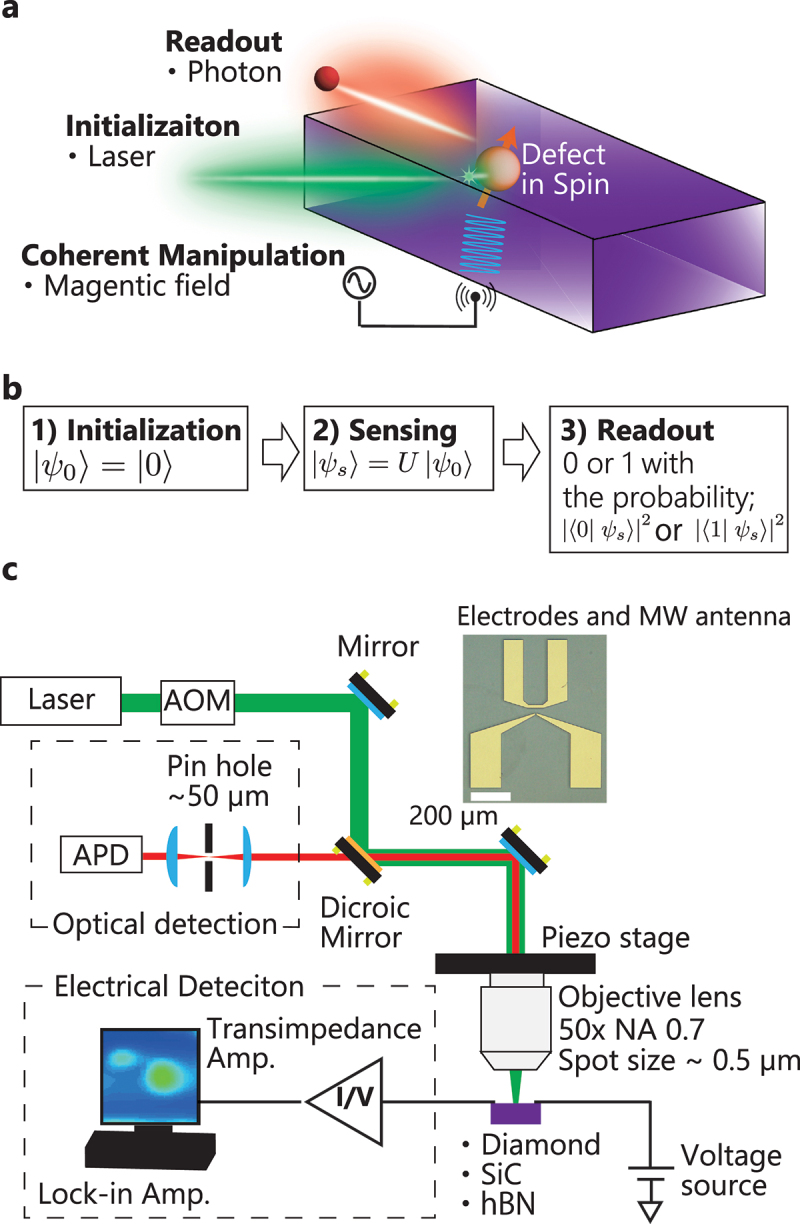
**a** Operation of a solid-state quantum sensor, encompassing the initialization of the spin, coherent manipulation and the readout of a defect spin. **b** typical quantum sensing protocol. **c** schematic illustrations of confocal laser microscopy and scanning photocurrent microscopy, demonstrating quantum sensing using optical and photoelectrical detection, respectively. The upper-right panel shows a photograph of the U-shaped microwave antenna for spin manipulation and two opposing electrodes for photoelectrical detection, separated by a gap of ∼10 μm. Scale bar: 200 μm.

The operation of all solid-state quantum sensors is predicated on three fundamental steps: (1) The preparation of a well-defined quantum state (e.g. spin initialization via thermal polarization or optical pumping), (2) sensing via spin manipulation interacting with external fields and (3) readout of the final quantum state (Figure 1(**a**),1(**b**)). Spin control frequently utilizes magnetic resonance techniques. For NV centers in diamond and defects such as V${\;}_{\mathrm{Si}}$ in SiC, the readout is typically optical or photoelectrical. Figure 1(**c**) illustrates representative quantum sensing implementations based on optical and photoelectrical detection schemes using confocal laser scanning microscopy and scanning photocurrent microscopy, respectively. A focused laser is used for spin initialization and readout, either optically via fluorescence or electrically via photocurrent. The objective lens with the magnitude of 50 and NA of 0.7 focuses the excitation beam onto a single or small ensemble of defects with a nearly diffraction-limited spot diameter of ∼0.5 μm. The focal position is raster-scanned using a piezoelectric stage, providing a typical travel range of up to 100 μm along the x, y, and z directions. The upper left region schematically shows integrated electrodes for electrical readout and a microwave antenna for coherent spin manipulation. The gap between the electrodes are ∼10 μm. In the optical detection scheme, fluorescence emitted from the defect is collected through a pinhole (diameter ∼50 μm) and detected using an avalanche photodiode (APD). In contrast, for photoelectrical detection, a constant bias voltage is applied across the electrodes, and the laser-induced photocurrent is measured using a transimpedance amplifier followed by lock-in detection.

Diamond is known to host a variety of defects and impurities that are advantageous for quantum devices. Examples of such centers include the NV center [4], the ST1 center [29] and the NiV center [30]. Among these candidates, the NV center is considered the most promising for solid-state quantum sensing due to the extremely long coherence times among solid-state electron spins [12,31]. To comprehend the operational mechanisms of the NV quantum center, the spin Hamiltonian $\left(H\right)$ can be delineated by the following equation:

(1) $$H=S^{T}\cdot D\cdot S+g_{e}\mu_{B}S\cdot B_{0},$$

The first term is described as the zero-field splitting (ZFS) term of the NV electron spin (S = 1), which depends on the electric field, temperature and pressure [4]. the ZFS parameters of *D* and *E* are defined as $D\;\equiv \;2D_{\mathrm{zz}}/3\;∼$ 2.87 GHz, and $E\;\equiv \;\left(D_{\mathrm{xx}}+D_{\mathrm{yy}}\right)/2$, respectively. The second term describes the Zeeman interaction of NV electron spins with a magnetic field, $g_{e}∼$ 2.003 and $\mu_{B}$ are g-factor of the NV electron spin and the Bohr magneton, respectively. Consequently, the diamond NV quantum sensor has the capacity to measure magnetic fields, electric fields, temperature and pressure.

SiC is known to harbor a variety of defects that are for utilization in quantum devices [32,33]. Among of them, V${\;}_{\mathrm{Si}}$ is the most extensively studied defect. The defect has spin values of S = 3/2 (V${\;}_{\mathrm{Si}}$) with its respective spin Hamiltonian exhibiting a comparable character to that of the NV center in diamond [34,35]. The effect of spin value on magnetic field measurement is discussed in Section 3.3. Since V${\;}_{\mathrm{Si}}$ is a single vacancy, unlike NV centers which possess four quantization axes, its response to the measured magnetic field is simpler. However, vector measurements require complex protocols [36]. A significant discrepancy has been observed in their ZFS values: V${\;}_{\mathrm{Si}}$ has a ZFS of approximately 35 MHz [37]. Although not discussed in this paper, in magnetic field measurements using level anti-crossing, this small ZFS also means that the required external magnetic field can be reduced by at least one order of magnitude compared to NV center [38].

In addition to bulk hosts such as diamond and SiC, color centers in hBN have recently attracted considerable interest as an alternative platform for solid-state quantum sensing. Owing to its atomically thin, van der Waals layered structure, hBN enables defect spins to be positioned within a few nanometers – or even sub-nanometer distances – from target systems. Such extreme proximity is particularly advantageous for sensing weak magnetic or electric fields from low-dimensional materials, molecular systems, and surface-bound phenomena, where the detectable signal strength scales strongly with sensor-sample distance. Several optically addressable spin defects, including boron-vacancy (V${\;}_{B}$) centers, have demonstrated room-temperature spin polarization, microwave manipulation, and ODMR-based readout. The two-dimensional geometry further offers opportunities for integration with heterogeneous material stacks and nanofabricated devices, suggesting potential routes toward ultrathin and flexible quantum sensors. At present, however, the technological maturity of hBN-based spin defects remains lower than that of diamond and SiC. In particular, reproducible defect creation, spectral stability, and spin coherence times are still under active development, and electrical readout schemes such as PDMR have not yet reached the same level of implementation as in bulk semiconductor hosts. Consequently, while hBN represents a promising future platform for surface-proximal and two-dimensional sensing, the present review focuses primarily on diamond- and SiC-based systems, where established optical and photoelectrical readout techniques already enable practical scanning-probe operation.

Table 2 summarizes representative material properties of solid-state spin-defect platforms, including the NV center in diamond, the V${\;}_{\mathrm{Si}}$ in SiC, and the V${\;}_{B}$ center in hBN. These parameters collectively determine the achievable spin-readout contrast, coherence time, optical efficiency, and ultimately the magnetic-field sensitivity attainable in bulk crystals and device implementations. Among these platforms, diamond NV centers currently provide the most balanced overall performance. Although the Debye-Waller factor is relatively small (∼3%) [39], NV centers exhibit high fluorescence count rates, large ODMR contrast (up to ∼40%), and long room-temperature coherence times [12]. In isotopically purified diamond, millisecond-scale $T_{2}$ times have been achieved, and further improvements can be realized through optimized material engineering and donor control [12]. Together with excellent thermal stability and mature single-crystal growth technology, these attributes establish diamond as the current benchmark platform for high-sensitivity quantum sensing and a widely adopted material for scanning-probe implementations.

**Table 2.** Comparison of various material properties of color center in diamond, SiC, and hBN.

Materials	NV center in Diamond	V${\;}_{\mathrm{Si}}$ in SiC	V${\;}_{B}$ in hBN
Zero-Phonon Line fraction (Debye-Waller factor) (%) at low temperature	∼ 3 [39]	6–9 [40,41]	
Fluorescence count rate (kcps)	∼ 250 [12]	∼ 10 [42]	
$T_{2}$ at room temperature Single in Natural Abundance (μs)	∼ 650 [43]	> 200 [42]	
Single in isotope pure (ms)	≈ 2.4 [12]		
Ensemble in natural abundance (μs)	≈ 700 [44]	∼ 50 [45]	2 [46]
Ensemble in isotope pure (μs)	≈ 700 [44]	∼ 100 [47]	0.1–0.2 [48]
Thermal Stability of defects (${\;}^{\circ }$C)	∼ 1130 [49]	∼ 600 [47,50,51]	∼ 500 [52]
Maturity of crystal growth			
		8 inch (commercialized)	
Wafer size	92 mm diameter [53]	12 inch (development)	
Dislocation Density (cm${\;}^{-2}$)	$10^{3}$ - $10^{5}$ [53,54]	10 ${\;}^{3}-10^{4}$ [55–58]	
		10	
Rocking Curve FWHM (arcsec)	5–300 [53]	Reaching the theoretical limit [56]	
		2–6	
Rabi Contrast (%)	∼ 37 [12]	(including ODMR Contrast) [42,59,60]	0.5–3 [46,61]
Shot-noise limited sensitivity with an optical detection Single DC (T/$\sqrt{\mathrm{Hz}}$)	∼ 6 × 10${\;}^{-9}$ [12]		
Ensemble DC (T/$\sqrt{\mathrm{Hz}}$)	460 × 10${\;}^{-15}$ [13]	4 × 10${\;}^{-9}$ [62]	2.87 $\times 10^{-6}$ [63]
Single AC (T/$\sqrt{\mathrm{Hz}}$)	∼ 9.1 × 10${\;}^{-9}$ [12]	358 × 10${\;}^{-6}$ [64]	
Ensemble AC (T/$\sqrt{\mathrm{Hz}}$)	210 × 10${\;}^{-15}$ [13]	(16–25) × 10${\;}^{-9}$ [62]	1 $\times 10^{-6}$ [65]
Sensitivity with a photoelectrical detection			
Ensemble DC (T/$\sqrt{\mathrm{Hz}}$)	1 × 10${\;}^{-7}$ [114]		
Ensemble AC (T/$\sqrt{\mathrm{Hz}}$)	2.9 × 10${\;}^{-8}$ [106]		

SiC hosts silicon-vacancy centers and offers a technologically mature semiconductor platform. The availability of large-diameter wafers and relatively low dislocation densities make SiC promising for mass production. However, lower photon count rates [42] and reduced ODMR contrast presently result in lower spin-readout performance compared with diamond-based systems [62]. These limitations mainly arise from the intrinsic optical and spin-dependent properties of V${\;}_{\mathrm{Si}}$ centers. Crystal can be an important factor for coherence properties such as $T_{2}^{*}$. However, the transition between $|m_{s}>=|+1/2>$ and |−1/2> of V ${\;}_{\mathrm{Si}}$ centers can be exploited as a strain-insensitive spin basis that exhibits $T_{2}^{*}$ much longer than the transitions between |±1/2> and |±3/2> and less sensitive to the electron-irradiation dose, indicating a reduced sensitivity to inhomogeneous broadening induced by local strain and crystal inhomogeneity [47]. Ongoing improvements in material purity, defect engineering, and photonic integration are expected to mitigate these limitations and enhance overall sensing performance.

Two-dimensional hBN has recently emerged as a promising host material due to its atomically thin geometry, which enables extreme proximity between the spin sensor and the target magnetic source. This feature is particularly advantageous for nanoscale and surface-sensitive scanning applications. At the same time, quantitative performance metrics – such as coherence time, ODMR contrast, and sensitivity – remain less mature than those of diamond and SiC, as reflected by the shorter coherence times and lower readout contrast summarized in Table 2. Defect reproducibility, spectral stability, and material uniformity therefore remain active research challenges.

Overall, the selection of the host material involves trade-offs among spin coherence, optical readout efficiency, fabrication scalability, and thermal robustness. Diamond remains the benchmark platform for high-sensitivity scanning magnetometry owing to its superior combination of coherence and optical readout performance, while SiC offers strong advantages for scalable semiconductor integration and hBN provides unique opportunities for ultrathin, surface-proximal sensing architectures.

## 3. Material-dependent spin initialization, manipulation, and optical readout

### 3.1. Initialization

Solid-state quantum sensors utilize electron and/or nuclear spins, with the initial step being spin polarization (initialization). The simplest and most fundamental spin polarization mechanism is thermal polarization, where the spin population follows the Boltzmann distribution. Although this mechanism describes the equilibrium spin population, it yields only below 1-% polarization at room temperature and typical magnetic fields. Therefore, thermal polarization is insufficient for high-sensitivity quantum sensing. Consequently, most modern solid-state quantum sensors, such as NV centers in diamond or V${\;}_{\mathrm{Si}}$ in SiC, employ active initialization methods of optical pumping to achieve near-unity polarization.

**Figure 2.**
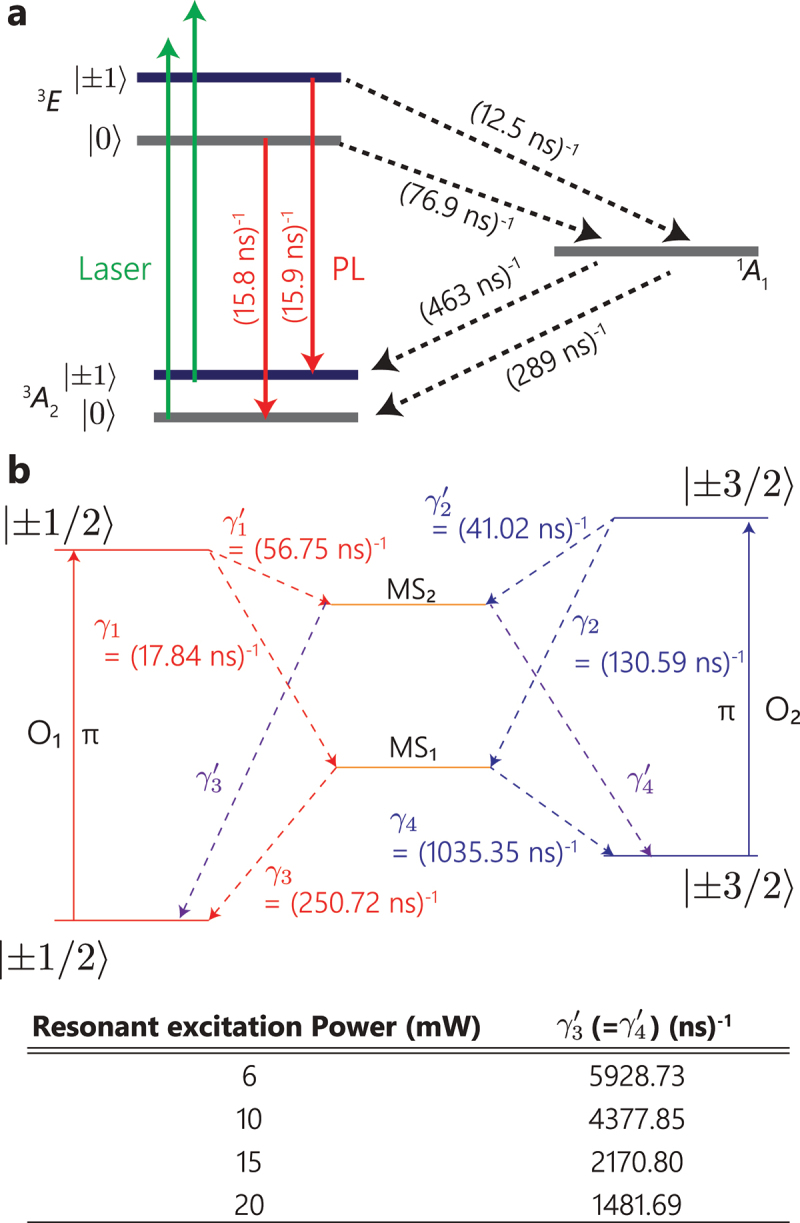
**a** Transition diagram of NV${\;}^{-}$ in diamond with transition rates. The solid and dashed down arrows represent the radiative and non-radiative transitions, respectively (see ref. [4]). **b** (top) the transition diagram of V${\;}_{\mathrm{Si}}$ (V2) with lifetimes (transition rates) measured at 5.5 K. (bottom) the lifetime of ($\gamma_{3{\;}^{\prime }}$‘and ($\gamma_{4{\;}^{\prime }}$’ as a function of the resonant excitation power (details are discussed in ref. [66]). Figure and table were adapted and modified from ref [66]. CC BY 4.0 [https://creativecommons.Org/licenses/by/4.0/)].

In contrast, the proposed and demonstrated quantum sensors based on NV centers in diamond and V${\;}_{\mathrm{Si}}$ in SiC can be initialized through optical pumping processes. This represents a substantial benefit for solid-state quantum sensors, as they are capable of functioning under ambient conditions over a broad temperature range. The subsequent section elucidates the mechanism of spin initialization for NV and V${\;}_{\mathrm{Si}}$ electron spins via optical pumping [4,66]. As illustrated in Figure 2(a), the energy levels of an NV center are depicted without a static magnetic field. Following laser excitation, an electron in the ground state (GS) (${\;}^{3}A_{2}$) is excited to the excited state (ES) (${\;}^{3}E$). The electron subsequently undergoes a relaxation process, either radiatively, returning to the ${\;}^{3}A_{2}$ state (illustrated by red solid arrows) or non-radiatively, via a metastable state (${\;}^{1}A_{1}$) (depicted by black dashed arrows). These relaxation pathways are spin dependent: when the NV electron spin is $|m_{s}>=|0>$, relaxation is primarily radiative. Conversely, for $|m_{s}>=|\pm 1>$, the electron preferentially relaxes through the non-radiative pathway. Once an electron relaxes to the ${\;}^{1}A_{1}$ state, it predominantly relaxes to the |0> in the ${\;}^{3}A_{2}$ state. The electron spin can be initialized into the |0> state with a high degree of fidelity (i.e. 90 % or more) by repeating the cycle of photoexcitation and relaxation.

V${\;}_{\mathrm{Si}}$ is classified as a spin defect with S = 3/2 [34] and it has been observed that GS and ES possess spin sublevels $|m_{s}>$ = |±1/2> and |±3/2>, respectively. The transition diagram of V${\;}_{\mathrm{Si}}$ (V2) is illustrated in Figure 2(**b**) [66]. As observed in the NV center in diamond, following photoexcitation (O${\;}_{1}$ or O${\;}_{2}$), an electron undergoes either a direct transition with photoemission or a non-radiative transition via metastable states (MS ${\;}_{1}$ or MS ${\;}_{2}$) to the GS. For initialization by laser irradiation, the transition probability of each transition, including the metastable states, is important. The lifetimes (transition rates) were measured at 5.5 K, as shown in Figure 2(**b**) [66]. The lifetimes from MS ${\;}_{2}$ to the GS ($\gamma_{3{\;}^{\prime }}$, $\gamma_{4{\;}^{\prime }}$) are very long compared to those from MS ${\;}_{1}$ ($\gamma_{3}$, $\gamma_{4}$) and the former show laser power dependence. Because of $1/\gamma_{3}<1/\gamma_{4}$, the transition tends to be preferentially towards |±1/2>. Since $\gamma_{3{\;}^{\prime }}$ (=$\gamma_{4{\;}^{\prime }}$) becomes large under high-laser-power conditions, the spin polarization after initialization shows laser power dependence.

### 3.2. Optical readout

Optically detected magnetic resonance (ODMR) of NV centers is a well-established technique in which spin-dependent intersystem crossing leads to spin-dependent fluorescence contrast under microwave excitation. The basic energy-level structure and readout mechanism have been extensively reviewed elsewhere [4] and are not repeated here. Instead, we focus on how materials properties influence the achievable ODMR contrast. The ODMR contrast of single NV centers directly reflects the intrinsic spin and charge properties of the defect, whereas ensemble measurements are additionally affected by dipolar interactions, inhomogeneous broadening, and orientation averaging. Consequently, single-defect contrast provides a clearer metric for evaluating material quality. NV centers can be created either by nitrogen ion implantation followed by annealing or during CVD growth. Ion implantation typically introduces vacancy clusters and complex defect structures near the NV center, which degrade spin coherence and charge stability, resulting in reduced ODMR contrast compared with high-purity CVD-grown material [67,68]. Charge-state engineering further plays a critical role. Incorporation of phosphorus donors during CVD growth stabilizes the NV${\;}^{-}$ charge state, increasing the steady-state NV${\;}^{-}$ population under optical excitation. Under such conditions, nearly 100% NV${\;}^{-}$ occupancy has been reported [69], compared with ∼50–70% in intrinsic diamond [70]. Using *n*-type phosphorus-doped diamond, single-NV ODMR contrasts as high as ∼37% have been achieved [12]. Surface termination and near-surface defects also strongly influence ODMR performance by affecting both charge stability and spin coherence through electric and magnetic noise sources [71]. These observations highlight that ODMR contrast is governed not only by the intrinsic defect physics but also by materials quality and device processing.

The principle of ODMR for the SiC V${\;}_{\mathrm{Si}}$ defect is analogous to that of the NV center, but its S = 3/2 spin makes the situation more complex. The application of a magnetic field in the direction of the c-axis gives rise to the splitting of each Kramers doublet because of the Zeeman effect, thereby yielding two observable transitions (|−1/2>↔|−3/2> and |+1/2>↔|+3/2>) [72], where. the ODMR contrast is reduced by half due to the presence of two distinct populations. In the case of fields misaligned with the c-axis, up to six ODMR signals can be detected.

The decrease in the ODMR contrast in the presence of a magnetic field is attributable to the S = 3/2 configuration. This is not exclusive to the contrast and the signal intensity also diminishes in pulse measurements such as Rabi, $T_{1}$ and $T_{2}$ measurements. To overcome this disadvantage, the measurements were performed on each of the two split signals (in the case of a parallel magnetic field) and the signals obtained were summed. This process has been shown to restore the signal intensity to its original value in the absence of a magnetic field [66,73].

### 3.3. Manipulation

#### 3.3.1. Continuous wave magnetic resonance

The continuous-wave magnetic resonance, including the ODMR and PDMR protocols, exhibits sensitivity to magnetic fields with a frequency in the DC ∼ kHz range. In this section, the NV quantum sensor is employed as a case study, given its analogous functionality to the SiC quantum sensor. The Hamiltonian of the NV center, in the presence of a magnetic field $B_{z}$ along the NV axis is

(2) $$H^{I}=D_{\mathrm{GS}}S_{z}^{2}+\gamma_{e}B_{z}S_{z}.$$

where $D_{\mathrm{GS}}$ is the ZFS, $S_{z}$ is the z component of the electronic spin and $\gamma_{e}=g_{e}\mu_{B}$ is the gyromagnetic ratio of the NV center. The resulting Zeeman splitting leads to resonance frequencies

(3) $$\omega_{\pm }=D_{\mathrm{GS}}\pm \gamma_{e}B_{z}.$$

The lifted energy levels are measured as follows: the continuous illumination of a laser excites the electronic state, which subsequently undergoes relaxation to the |0> state. Simultaneous irradiation with the continuous microwaves whose magnetic field component is perpendicular to the NV axis has been shown to induce transitions between the |0> and |±1> states, provided that the microwave frequency, ω, matches either of the resonant frequencies, $\omega_{\pm }$ (see further details in Sec. 3.3.2). Consequently, the resonant frequencies, $\omega_{\pm }$, can be determined by observing the reduction of fluorescence in ODMR or the change in the photocurrent in PDMR.

While the basic detection mechanism is well established, the achievable CW magnetic-field sensitivity is strongly governed by material properties. To first order, the sensitivity can be approximated as

(4) $$\eta_{\mathrm{CW}}\propto \frac{\Delta \nu }{C\sqrt{R}},$$

where Δν is the resonance linewidth, C is the ODMR contrast, and R is the detected photon rate [1,2]. All three parameters are highly sensitive to crystal growth method, defect density, strain homogeneity, and charge-state stability.

The linewidth Δν is fundamentally limited by the inhomogeneous dephasing time $T_{2}^{*}$, but in ensemble measurements it is often dominated by spatial variations in lattice strain and defect-induced electric-field fluctuations. Recent advances demonstrate that CW-based NV magnetometry can approach the pT/$\sqrt{\mathrm{Hz}}$ regime when employing a narrow-linewidth ensemble (∼28 kHz) with long dephasing time ($T_{2}^{*}\;∼$ 8.5 μs) and optimized optical readout [74]. Such narrow linewidths are achievable only in high-quality CVD-grown diamond with excellent strain homogeneity and low residual defect density. In contrast, strain inhomogeneity significantly broadens the ODMR linewidth. For example, in 10-MeV electron-irradiated HPHT diamond, irradiation-induced vacancy/interstitial complexes generate spatially varying lattice strain, leading to MHz-scale broadening; a maximal ODMR splitting of ∼6 MHz was observed at high fluence (≈ 10${\;}^{18}$ cm${\;}^{-2}$) [75]. These results directly illustrate that CW-ODMR performance is strongly governed by crystal growth conditions and defect engineering.

In SiC, similar material-dependent behavior has been observed for V${\;}_{\mathrm{Si}}$ defects. For DC magnetic-field sensing, the nuclear-spin environment strongly limits the inhomogeneous dephasing time $T_{2}^{*}$. Isotope purification (0.15% ${\;}^{13}$C and 0.01% ${\;}^{29}$Si) has been shown to increase $T_{2}^{*}$ at room temperature by approximately one order of magnitude, from ∼ 0.4 μs to ∼ 4 μs in the {±1/2,±3/2} spin basis [47]. Furthermore, the silicon-vacancy center in SiC provides an alternative spin basis {−1/2,+1/2} that is largely disentangled from the zero-field splitting and therefore relatively insensitive to strain. In isotopically purified material, this strain-insensitive basis has demonstrated $T_{2}^{*}\approx$ 20 μs [47]. These material improvements have enabled DC magnetic-field sensitivities down to ∼ 4 nT/$\sqrt{\mathrm{Hz}}$ [62], highlighting the critical role of isotope engineering and defect-basis selection in determining CW sensing performance.

#### 3.3.2. Rabi oscillation

The Rabi measurement quantifies the amplitude and frequency of oscillating magnetic fields [76,77]. The Hamiltonian with static $B_{z}$ and oscillating $B_{x}$ with a frequency ω is

(5) $$H^{\mathrm{II}}=D_{\mathrm{gs}}S_{z}^{2}+\gamma_{e}B_{z}S_{z}+\gamma_{e}B_{x}\cos\left(\mathrm{\omega t}\right)S_{x},$$

where $S_{x}$ is the x component of the electronic spin. When the resonant condition $\omega ∼\omega_{+}$ is met, the Hamiltonian on the rotating frame can be simplified to the following form:

(6) $$H_{rot}^{II}=\frac{\Omega }{2}\left(|0><1|.+|1>.<0|\right),$$

where $\gamma_{e}B_{x}$ is denoted as Ω. The time evolution of the initial state |0> is expressed as

(7) $$|\psi^{\mathrm{II}}(t)>=\cos\left(\frac{\Omega }{2}t\right)|0>+i\sin\left(\frac{\Omega }{2}t\right)|1>,$$

which leads to the oscillating probability observing |0>:

(8) $$p_{|0>}^{II}(t)=\frac{1}{2}+\frac{1}{2}\cos\left(\mathrm{\Omega t}\right).$$

This equation suggests that both the amplitude $B_{x}$ and frequency ω of the magnetic field can be obtained when the resonant frequency ω is tuned to $∼\omega_{+}$. The pulse which brings |0> into $|+>=\left(|0>+|1>\right)\sqrt{2}$ is called a π/2-pulse, whereas the pulse which brings |0> into |1> is called a π-pulse. These pulses are used in the following sensing protocols.

In practice, Rabi fringes decay over time, and both the achievable contrast and the effective decay time are strongly influenced by material properties. The Rabi contrast depends critically on stable spin initialization and readout. Donor doping can stabilize the negative charge state and suppress paramagnetic defect formation, thereby enhancing the oscillation amplitude. For example, a Rabi contrast as high as 37% has been reported in donor-engineered diamond [12], demonstrating the direct impact of defect and charge-state control on coherent manipulation fidelity. In ensemble measurements, the apparent Rabi decay is often dominated not by intrinsic relaxation but by a spatial distribution of the Rabi frequency Ω across the sensing volume. Such inhomogeneity arises from variations in microwave-field strength, defect density, and strain. This effect becomes particularly significant in GHz-field sensing, where ensemble inhomogeneous linewidth and microwave-field gradients directly limit performance. Heterodyne detection schemes have been proposed to mitigate this limitation and effectively extend the observable Rabi decay time [78,79]. In bulk diamond containing four crystallographic NV orientations, only a subset of centers contributes constructively under a given bias field configuration. Preferential alignment during CVD growth reduces orientation averaging and significantly improves ensemble Rabi contrast [80–82]. These observations indicate that Rabi-based sensing performance is governed not only by intrinsic spin coherence but also by charge engineering, microwave-field uniformity, and crystallographic control.

#### 3.3.3. Ramsey interferometry

The Ramsey interferometry is sensitive to the energy splitting ω because of the accumulation of the relative phase between |0> and |1> [83]. In the first step of the protocol, the state is prepared to be the sensing state $|\psi_{s}^{III\;}>$ upon the application of a π/2-pulse ($R_{\pi }/2$) to the initial state of |0>:

(9) $$|\psi_{s}^{III}>=R_{\pi /2}|0>=|+>=\frac{|0>+|1>}{\sqrt{2}},$$

Subsequently, the sensing state acquires a phase under the Hamiltonian $H^{I}$ within a duration of t:

(10) $$|\psi_{s{\;}^{\prime }}^{\mathrm{III}}>=\frac{|0>+e^{-i\omega_{+}t}|1>}{\sqrt{2}}.$$

Using a second π/2-pulse, the relative phase is mapped onto the measurable population difference:

$$|\psi_{m}>=R_{\pi /2}|\psi_{s{\;}^{\prime }}^{\mathrm{III}}>$$

(11) $$=\frac{\left(1+e^{-i\omega_{-}t}\right)|0>+\left(1-e^{-i\omega_{+}t}\right)|1>}{\sqrt{2}}.$$

The probability of observing |0> is

(12) $$p_{|0>}^{III}(t)=\frac{1}{2}+\frac{1}{2}\cos\left(\mathrm{\omega t}\right),$$

from which the amplitude of the target magnetic field can be obtained as the sine fringe frequency.

In Ramsey magnetometry, the DC magnetic-field sensitivity scales approximately as

(13) $$\eta_{\mathrm{Ramsey}}\propto \frac{1}{\gamma_{e}C\sqrt{NT_{2}^{*}}},$$

indicating that improvements in the inhomogeneous dephasing time $T_{2}^{*}$ directly translate into enhanced sensitivity [1,2].

In diamond, the Ramsey linewidth is primarily governed by the ${\;}^{13}$C nuclear spin bath and paramagnetic impurities. Its dependence on ${\;}^{13}$C concentration has been extensively studied, showing that $T_{2}^{*}$ can reach ∼ 30 μs at a reduced ${\;}^{13}$C abundance of 0.03% [43,84]. Further improvement can be achieved through controlled phosphorus donor doping, where donated electrons suppress the formation of paramagnetic vacancy complexes; under optimized conditions, $T_{2}^{*}$ values approaching ∼ 1.5 ms have been reported [12]. For ensembles, $T_{2}^{*}$ is often limited by nitrogen-related paramagnetic defects. Systematic studies indicate that $T_{2}^{*}$ spans approximately 0.1–10 μs for nitrogen concentrations in the 1–50 ppm range at room temperature [44]. For shallow NV centers, additional degradation arises from surface band bending and termination chemistry, which affect charge stability and introduce electric and magnetic noise. With appropriate donor doping, near-surface NV${\;}^{-}$ centers (∼ 5 nm depth) with $T_{2}^{*}\approx 0.81\;\mu$s have been demonstrated [85].

Similar material-dependent limitations are observed in SiC-based defects. In V${\;}_{\mathrm{Si}}$ centers, the nuclear spin environment strongly influences $T_{2}^{*}$. Isotope purification (0.15% ${\;}^{13}$C and 0.01% ${\;}^{29}$Si) increases $T_{2}^{*}$ at room temperature by approximately one order of magnitude, from ∼ 0.4 μs to ∼ 4 μs in the {±1/2,±3/2} basis [47]. Moreover, the strain-insensitive {−1/2,+1/2} spin basis enables further improvement to $T_{2}^{*}\approx$ 20 μs in isotopically purified samples [47]. These advances have enabled DC magnetic-field sensitivities down to ∼ 4 nT/$\sqrt{\mathrm{Hz}}$ [62]. Overall, Ramsey magnetometry directly converts materials improvements in nuclear-spin purification, defect control, and surface engineering into enhanced DC sensitivity, making $T_{2}^{*}$ a key figure of merit for comparing diamond and SiC quantum sensors.

#### 3.3.4. Spin echo

The fundamental premise of the spin echo protocol is the cancellation of the DC magnetic field noise by applying a π-pulse in the middle of the Ramsey measurement. A DC magnetic field is canceled over the echo sequence because the first and second phase accumulations have opposite signs. In other words, if the magnetic field signal is synchronized with the sequence, the phase proportional to $B_{z}$ is accumulated.

The spin-echo protocol suppresses quasi-static magnetic-field noise and reveals the intrinsic coherence time $T_{2}$ of the defect spin. The achievable AC magnetic-field sensitivity therefore scales approximately as

(14) $$\eta_{\mathrm{Echo}}\propto \frac{1}{\gamma_{e}C\sqrt{NT_{2}}},$$

making $T_{2}$ the key materials-dependent figure of merit in echo-based sensing [1,2].

In diamond, the echo decay is governed primarily by coherent coupling between the NV electron spin and the surrounding ${\;}^{13}$C nuclear-spin bath [86]. Systematic studies show that $T_{2}$ spans from ∼ 10 μs to 1.8 ms as the ${\;}^{13}$C concentration is reduced from ∼ 10% to ∼ 0.3%, exhibiting an approximately inverse dependence on nuclear-spin density [43,84]. Phosphorus donor doping can further extend $T_{2}$ to ∼ 2.4 ms by suppressing paramagnetic vacancy complexes [12]. For near-surface NV centers, electric and magnetic surface noise becomes increasingly important; however, recent work demonstrates that surface-induced strain engineering combined with weak DC magnetic fields can significantly enhance coherence even for ∼ 1 nm-deep NV centers in ${\;}^{12}$C-enriched diamond [87]. These results illustrate that nuclear-spin purification, defect control, and surface engineering directly determine the achievable echo coherence.

Similar material-dependent behavior is observed in SiC-based spin defects. In natural-abundance 4 H-SiC, single V${\;}_{\mathrm{Si}}$ centers exhibit echo signals persisting for at least 200 μs at room temperature [42]. Ensemble measurements report $T_{2}=47\pm 8\;\mu$s [45]. Theoretically, millisecond-scale $T_{2}$ is expected in SiC due to reduced nuclear-spin flip-flop interactions arising from the binary lattice structure and the energetic separation of ${\;}^{29}$Si and ${\;}^{13}$C spin baths at moderate magnetic fields ($(B\underset{\text{\textbackslash{}\textasciitilde{}}}{\;>}\;\;x300\;G)$ [88,89]. Consistent with these predictions, $T_{2}$ values of 1.2–1.3 ms have been experimentally demonstrated for ensemble divacancies in natural-abundance SiC at 20 K [90].

Overall, echo-based sensing directly converts improvements in nuclear-spin environment and defect engineering into extended $T_{2}$, thereby enhancing AC magnetic-field sensitivity. Comparison of diamond and SiC indicates that both platforms can reach millisecond-scale coherence under optimized conditions, though via distinct materials mechanisms.

#### 3.3.5. Dynamical decoupling

As an extension of the echo sequence, a narrower bandwidth of the target signal frequency can be obtained using dynamic decoupling (DD) protocols [91]. The DD sequence is comprised of a train of π-pulses which are equally spaced by τ, thereby suppressing noises at all frequencies except for the harmonics of τ. The DD sequence has been subject to several variations, including Carr-Purcell-Meiboom-Gill (CPMG) [92,93], periodic dynamical decoupling (PDD) [94] and XY8 [95]. Because these sequences are insensitive to magnetic noise other than the harmonics of τ, the coherence time can be extended significantly. However, the achievable effective coherence time to $T_{2}^{DD}$ remains fundamentally limited by materials-dependent factors. Pulse imperfections arising from microwave-field inhomogeneity, dielectric loss, and heating become increasingly important at large pulse numbers. In high-defect-density samples, residual magnetic noise from paramagnetic impurities limits the ultimate extension of coherence. Thus, improvements in nuclear-spin purification, defect density reduction, and microwave engineering are essential for realizing the full potential of DD protocols. Moreover, the tunable frequency response of DD sequences enables noise spectroscopy, providing a powerful tool to characterize spin impurities and materials quality in diamond and SiC [45,96].

#### 3.3.6. Quantum heterodyne detection

Quantum heterodyne detection (Qdyne) has been demonstrated to enable ultrahigh-frequency resolution that exceeds the limit set by the coherence time of the color center. Qdyne utilizes a stroboscopic (under-sampling) principle, whereby the phase of the fluorescence signal is recorded at fixed, equally spaced time intervals. This sampling technique has been shown to cause aliasing, thereby enabling the accumulation of phase shifts to accurately determine the frequency of the target oscillating signal [97,98]. Although Qdyne overcomes the coherence-limited frequency resolution of conventional pulsed sensing, the signal-to-noise ratio per unit time remains governed by materials-dependent parameters such as the contrast C, coherence time $T_{2}$, and spin-relaxation time $T_{1}$. Long-term frequency stability further requires suppression of charge-state fluctuations, thermal drift, and defect-induced noise. Consequently, even in advanced protocols such as Qdyne or coherently averaged synchronized readout (CASR), ultimate performance is bounded by spin coherence, relaxation, and materials stability. These considerations highlight that while sophisticated control protocols can surpass apparent coherence limits in frequency resolution, their practical sensitivity and stability remain intrinsically tied to materials engineering.

### 3.4. Sensing performance

In the domain of quantum sensing, critical performance metrics encompass the target signal frequency, sensitivity, frequency resolution and dynamic range. It is generally accepted that protocols with a higher target signal frequency offer enhanced sensitivity yet concomitantly exhibit diminished frequency resolution and dynamic range. Therefore, it is imperative to select an appropriate sensing protocol based on the target signal.

The signal whose frequency exceeds the optical transition rate of the NV center is potentially difficult to detect, but a higher frequency is still detectable by pulsed sequences. The CW ODMR can detect a DC ∼ kHz signal. The Rabi can detect a DC ∼ GHz signal because the resonant condition occurs within the range of the ZFS order. The Ramsey can detect DC ∼ 100 kHz signals because the initialization takes a few microseconds in each repetition. The spin echo and DD have the capacity to detect frequencies of an ideal nature that are very high frequency, but in fact, this is limited to tens of MHz because the π-pulse takes ∼ 100 ns.

The sensitivity, defined in units of T/$\sqrt{\mathrm{Hz}}$, is a measure of the minimum detectable magnetic field within a given time. It depends on noise and the slope of the fluorescence signal with respect to the magnetic field. The noise is fundamentally limited by the photon shot noise and the spin projection noise. The slope of the curve depends on the signal contrast and linewidth. The signal contrast is higher in the pulsed sequence than in the CW-ODMR. In the CW ODMR spectra, the linewidth is limited by the coherence time $T_{2}^{*}$ but usually deteriorates with the MW power [99]. In the fringe of Ramsey, the linewidth is limited by only $T_{2}^{*}$. In the echo sequence, the coherence time is extended to $T_{2}$, resulting in better sensitivity than that of Ramsey [100]. DD sequence also extends the coherence time to $T_{2}^{DD}$, which gives an even better sensitivity [31,101].

The frequency resolution is converted to the bandwidth of the window functions. This is expressed in the following form [102] for the window function of the DD sequence that has equally τ spaced n pulses:

(15) $$W_{\mathrm{DD}}(\omega )=\frac{\sin\left(\frac{\omega }{2}\mathrm{n\tau}\right)}{\frac{\omega }{2}\mathrm{n\tau}}\tan\left(\frac{\omega }{2}\tau \right)\sin\left(\frac{\omega }{2}\mathrm{n\tau}\right).$$

The first sinc term is dominant at the frequency of 1/(2τ) and the peak has a bandwidth of 1/(nτ). Although increasing n narrows the bandwidth, the total interrogation time nτ is bounded by the coherence time $T_{2}^{DD}$, which is ultimately materials limited. Therefore, improvements in nuclear-spin environment and defect engineering directly enhance spectral resolution.

Consequently, increasing the pulse number n enables the DD sequence to achieve enhanced frequency resolution. In practice, however, the total sensing time nτ is fundamentally restricted by the coherence time discussed above, which in turn limits the achievable bandwidth. To circumvent this limitation, correlation spectroscopy probes the correlations between two DD sequences, allowing the bandwidth to reach $1/T_{1}$ [103]. Because $T_{1}$ is governed by phonon interactions and defect-related relaxation processes, materials engineering directly determines the achievable resolution. For example, $T_{1}$ can be extended from several milliseconds to the order of one second by cooling the system from room temperature to 77 K [31]. Ultimately, even narrower bandwidths approaching 1 Hz can be achieved in the CASR scheme [104], in which magnetometry sequences with spin readouts are synchronized to an external clock. Nevertheless, the long-term performance of such advanced schemes remains limited by spin-relaxation processes and materials stability.

The dynamic range of the detectable signal amplitude is subject to an trade-off with sensitivity. In CW-ODMR, relatively large magnetic fields can be detected directly. In other pulsed sequences, the oscillating signal fringe possesses a 2π ambiguity, thereby limiting the detectable range to one period of the fringe. Extending the sensing time improves sensitivity but reduces dynamic range, whereas shortening the sensing time increases range at the expense of precision. Since the maximum sensing time is fundamentally bounded by coherence, this trade-off is ultimately materials limited.

The trade-off between sensitivity and measurement range in the conventional pulsed sequences can be resolved by combining pulse sequences with different interpulse intervals and optimizing them via a Bayesian algorithm [105]. This approach enabled a dynamic range of nearly seven orders of magnitude for a single NV center at room temperature while preserving high sensitivity, which is the largest dynamic range ever demonstrated for NV centers [105]. Even in such optimized schemes, the achievable performance remains constrained by coherence time, readout contrast, and spin-relaxation processes determined by materials quality. Overall, while protocol choice determines the nominal frequency regime, the achievable sensitivity, frequency resolution, and dynamic range are fundamentally constrained by materials-dependent parameters including $T_{2}^{*}$, $T_{2}$, $T_{1}$, microwave homogeneity, strain, and defect density.

## 4. Photoelectrical readout of spin defects

### 4.1. Photoelectrical readout

**Figure 3.**
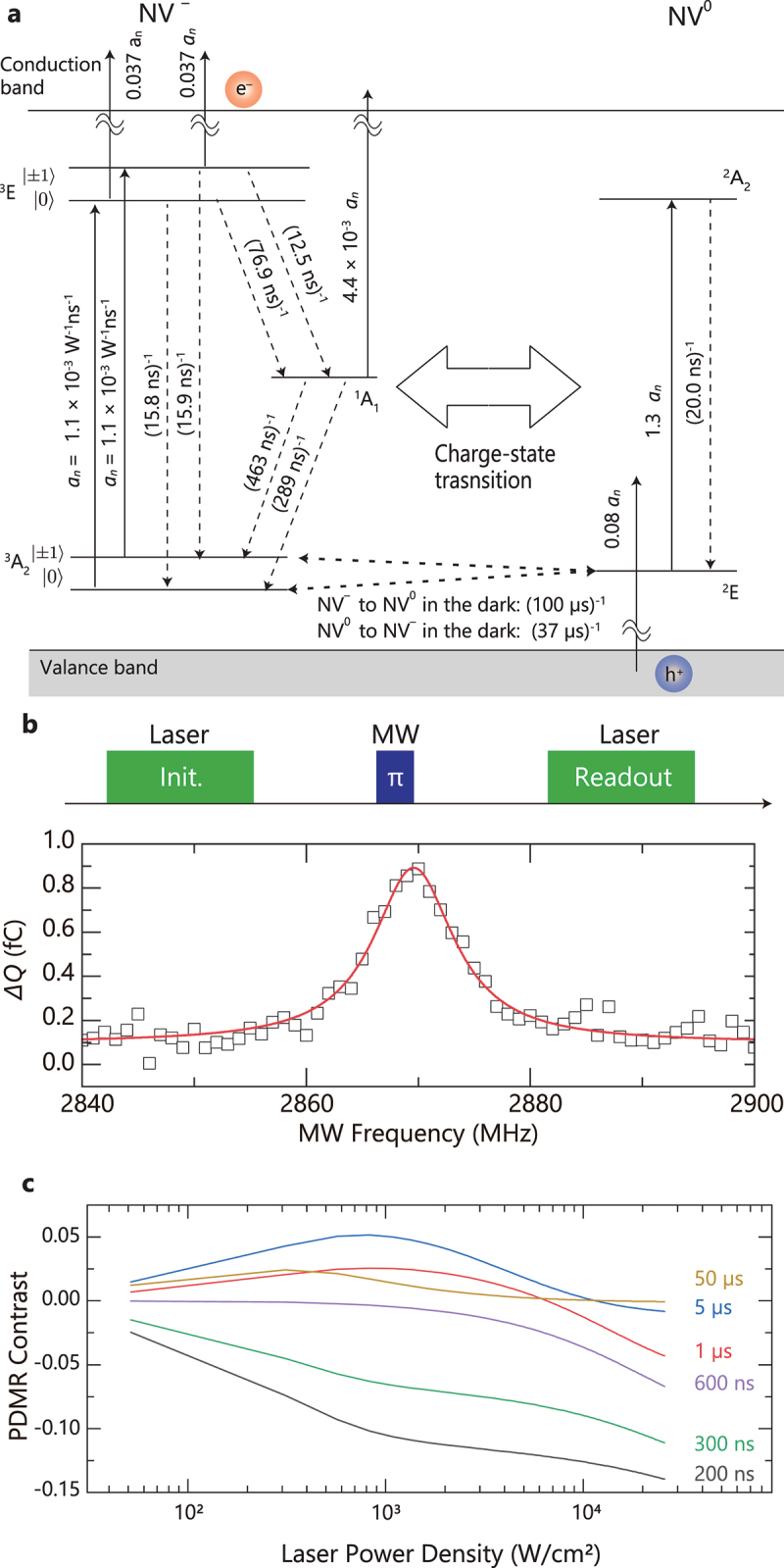
**a** Laser excitation and relaxation rates under the 532-nm laser illumination with the charge-state transition between NV${\;}^{-}$ and NV${\;}^{0}$. **b** PDMR spectrum of the NV center exhibits a positive contrast with the pulse sequence at the top. **c** PDMR contrast as a function of the laser power density with six different integration times under laser illumination. **a**, **b**, and **c** adapted with permission and modified from: reprinted figure with permission from [H. Morishita, N. Morioka, T. Nishikawa, H. Yao, S. Onoda, H. Abe, T. Ohshima, and N. Mizuochi. Spin-dependent dynamics of photocarrier generation in electrically detected nitrogen-vacancy-based quantum sensing. Phys. Rev. Appl. **19**, 034061 (2023).] copyright (2023) by the American physical Society.

PDMR detects the NV spin states by measuring the changes in the photocurrent generated under magnetic resonance. Under green laser excitation, electron-hole pairs are produced as the NV center undergoes charge-state conversion between NV${\;}^{-}$ and NV${\;}^{0}$. In contrast to the spin-to-charge conversion (SCC), the PDMR monitors spin-dependent ionization via photocurrent [14,106,107]. The overall photoelectrical detection process can be regarded as a sequence of consecutive steps comprising photoionization, transport and collection of the generated charge carriers, and electrical readout. At a conceptual level, for a fixed number of defects and excitation cycles, the number of detected charges $N_{\mathrm{detection}}$ can be expressed as

(16) $$N_{\mathrm{detection}}\propto P_{\mathrm{ionization}}\times \eta_{\mathrm{collection}}\times \eta_{\mathrm{readout}},$$

where $P_{\mathrm{ionization}}$ is the photoionization probability, $\eta_{\mathrm{collection}}$ is the efficiency with which the generated carriers are transported to and collected by the electrodes, and $\eta_{\mathrm{readout}}$ represents the effective electrical readout efficiency, including losses and bandwidth limitations in the readout circuitry. For PDMR, however, a large total number of detected charges does not necessarily correspond to a large magnetic-resonance signal. The spin-dependent component ($\Delta N_{\mathrm{PDMR}}$) can be described conceptually as

(17) $$\Delta N_{\mathrm{PDMR}}\propto C_{\mathrm{spin}}\times N_{\mathrm{detection}},$$

where $C_{\mathrm{spin}}$ represents the spin-dependent ionization contrast. Thus, even when the charge-collection efficiency approaches unity under appropriate bias and device conditions, the practical PDMR performance can remain limited by the photoionization probability and spin-dependent ionization contrast. The signal-to-noise ratio is further affected by dark current, spin-independent background photocurrent, current-amplifier noise, laser-intensity noise, charge-state fluctuations, and the local charge environment near the target defects. To describe these processes in more detail, we first consider the photocarrier-generation mechanism of the NV center under green-laser excitation, as illustrated in Figure 3(**a**). Furthermore, it elucidates the laser excitation and relaxation rates (illustrated by the solid and dashed arrows, respectively), as previously discussed in Ref [107]. In this process, an electron in the NV${\;}^{-}$ ${\;}^{3}A_{2}$ state is ${\;}^{3}E$ states and then to the conduction band (CB). Alternatively, an electron in ${\;}^{1}A_{1}$ state can be excited to the CB after relaxation from ${\;}^{3}E$ to ${\;}^{1}A_{1}$ state. This process of ionization produces a conduction electron and converts the NV${\;}^{-}$ to the NV${\;}^{0}$ state. The NV${\;}^{-}$ state can emit a hole into the valence band and recapturing an electron, thereby cycling back to NV${\;}^{0}$. This continuous cycle maintains steady photocarrier generation, enabling direct photoelectrical readout of the spin resonance. Although photoionization is accompanied by charge-state conversion during the readout process, the defect is continuously reinitialized under optical excitation, allowing repeated measurement cycles and signal accumulation. Consequently, coherent spin manipulation [60,107–112], spin-echo measurements [60,107,111–113], and nuclear-spin-related measurements [112–114] can be performed using PDMR in a manner analogous to ODMR. As outlined in Sec. 3.1, the NV electron spin can be initialized into the |0> state through the utilization of optical pumping. The occurrence of magnetic resonance results in the spin population transitioning to the |±1> state, impacting the way photocarriers are generated by the green laser. If the photoelectrons are predominantly excited from the ${\;}^{3}E$ state, the photocurrent undergoes a decrease under resonance, thereby yielding a negative contrast. Conversely, when excitation primarily occurs from the comparatively slower excitation from the long-lived ${\;}^{1}A_{1}$ state, the photocurrent increases, resulting in positive contrast. (Figure 3(**b**)). The predominant pathway is contingent on factors such as the excitation laser power, which exerts a substantial influence on the contrast sign [107] (Figure 3**c**). As demonstrated by Bourgeois et al. [14], the presence of acceptor-type defects has been shown to cause an increase in photocurrent during resonance, or positive contrast. Increasing the excitation power generally enhances the photoionization probability and the total photocurrent. However, this increase does not necessarily produce a proportional improvement in the spin-dependent PDMR signal. If the resonance-on and resonance-off photocurrents increase by the same relative factor, the PDMR contrast remains unchanged, while the photocurrent difference between the two conditions increases. Under shot-noise-limited conditions, this can improve the signal-to-noise ratio. More generally, however, the power dependence of the PDMR signal-to-noise ratio is determined by the relative power dependences of the spin-dependent signal, background photocurrent, and noise contributions arising from photocurrent shot noise, electronic readout noise, and laser-intensity fluctuations. Laser-intensity noise is not unique to PDMR and can also affect ODMR measurements. Consequently, to optimize PDMR sensitivity, it is imperative to meticulously manipulate the excitation conditions, laser wavelength and impurity or defect concentrations in the diamond. In comparison with optical techniques, photoelectrical detection represents a pivotal technology for the integration of quantum sensors, with the potential to achieve enhanced collection efficiency, thereby circumventing the optical limitations imposed by the high refractive index of diamond [107,110]. Utilization of PDMR, the detection of NV electron spin and coupled nuclear spins has been demonstrated [110,113], as has DC and AC magnetic sensing, which exhibit sensitivities of 100 nT/$\sqrt{\mathrm{Hz}}$ and 29 nT/$\sqrt{\mathrm{Hz}}$, respectively [107,115]. It is noteworthy that Hrubesch et al. [109] estimated that PDMR-based magnetometers could be approximately three times more sensitive than conventional ODMR, thereby highlighting the technique’s potential to advance the performance of NV quantum sensors.

**Figure 4.**
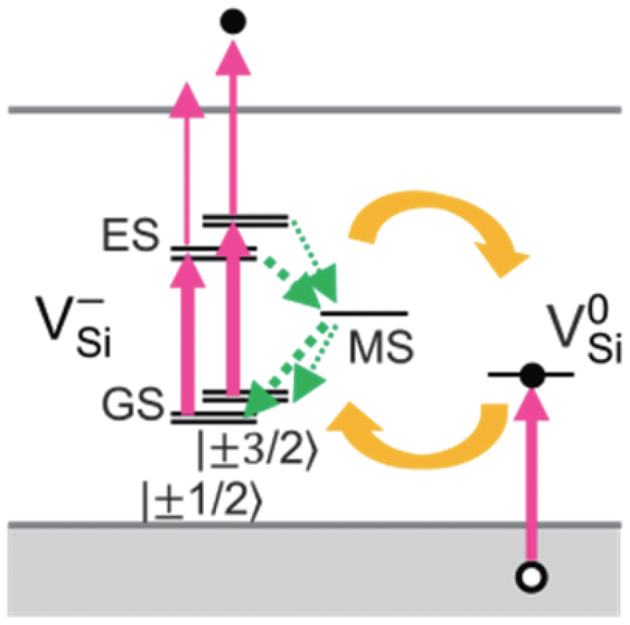
PDMR mechanism of the negatively-charged silicon vacancy.

**Figure 5.**
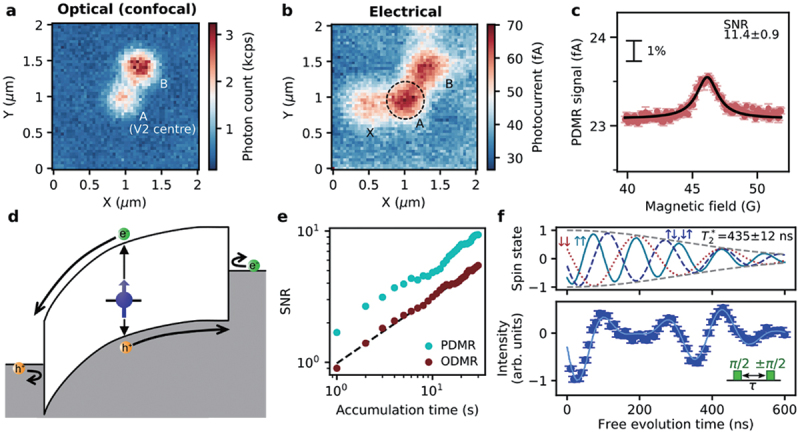
Photoelectrical detection of single silicon vacancies [60]. **a** fluorescence and **b** photocurrent scanning images of the same area in a 4 H-SiC PDMR device. Defect a is identified as a single V2 center and defect B is assumed to be a single V1 center. Spot X is an unidentified defect only observable using photoelectrical imaging. **c** magnetic-field sweep PDMR spectra of a V2 center at a fixed RF frequency of 199 MHz. The inset bar represents a signal contrast of 1 %. **d** schematic representation of a back-to-back Schottky diode under flat-band bias, facilitating concurrent efficient leakage blocking and photocurrent collection. **e** comparison of the SNR between ODMR and PDMR for defect A. The dashed black line indicates the shot-noise-limited SNR for ODMR. **f** PDMR-based Ramsey interferometry of defect A, demonstrating hyperfine coupling to two fourth-nearest-neighbor ${\;}^{29}$Si nuclear spins (upper panel: simulation, lower panel: experimental data). All panels were adapted from ref. [60] under CC BY 4.0 [https://creativecommons.Org/licenses/by/4.0/]; panels **c** and **e** were replotted for this review.

SiC is a material that has been extensively developed in the field of electronics. Due to its advanced device technologies, it is regarded as a promising platform for the sophisticated integration of quantum technologies and electronics. Consequently, the utilization of spin detection via PDMR in SiC is regarded as a highly promising technique. In SiC, PDMR has been demonstrated for the V2 electronic spin ensemble [111] and subsequently, the ENDOR-based detection of ${\;}^{29}$Si nuclear spins located at the second-nearest neighbor sites of the V2 center has also been reported [112]. Recently, the photoelectrical imaging and the single-spin detection of the single V2 center have been realized [60], as demonstrated in Figure 5(**a-c**). The realization of single-defect PDMR represents an important milestone for photoelectrical spin detection. Single-defect PDMR detection enables access to various fundamental properties necessary to optimize PDMR performance, such as signal intensities from a single defect, power-dependent ionization efficiency, the effects of other defects and background, and sequence strategies to reduce signals. This information deepens physical understanding, which is crucial to designing highly sensitive sensors in the future. Furthermore, single-defect PDMR can be combined with a scanning-probe sensing technique, and thus the single-defect PDMR is also important for practical quantum sensing applications. The mechanism of PDMR of the V2 center, as illustrated in Figure 4, is analogous to that of the NV center in diamond. The V2, the negatively charged state, absorbs two photons, resulting in the emission of a charge carrier into the energy bands. The question of whether the second photo absorption occurs while the system is in the excited-state quartet or in the metastable doublet state remains unresolved [111]. However, the observation of the positive contrast sign for single defects, the same as in ODMR, indicates that ionization predominantly occurs while the system resides in the quartet ES [60]. The combination of a properly biased back-to-back Schottky contact device (Figure 5(**d**); see Sec. 4.3) with laser pulse engineering [60,109] has been demonstrated to achieve a signal-to-noise ratio approximately 1.7 times higher than that of shot-noise-limited ODMR for the same defects (Figure 5(**e**)). This improvement arises from efficient charge collection in the photoelectrical scheme, compared with the limited photon collection efficiency inherent to ODMR. This enhancement approaches the ODMR enhancement achieved through the utilization of solid-immersion lenses; nevertheless, the adoption of more efficient ionization would further augment the SNR in PDMR. The improvement in PDMR sensitivity has enabled the use of coherent detection techniques for the readout of single defects, such as Ramsey interferometry (Figure 5(**f**)) and Hahn-echo, thereby evidencing clear nuclear spin coupling. Although the application of PDMR in SiC has been primarily focused on the detection of silicon vacancies, there is a possibility that its applicability may extend to a broader range of defects. While the optical technique is inherently limited by the sensitivity of photodetectors, the photoelectrical method provides a non-optical pathway that may allow for the detection of defects spanning a broader range of spectral characteristics, as seen in a recent study [60], revealing an additional unidentified feature in the photocurrent image (X in Figure 5**b**) that was not visible in the fluorescence image (Figure 5**a**). For such photocurrent-active defects, photoluminescence-based characterization can be difficult when the defects are non-emissive or weakly emissive, emit outside the efficient detection range of available photodetectors, or cannot be spectrally isolated due to overlapping emission from other centers. In such cases, wavelength-dependent photoionization responses can provide spectroscopic fingerprints that are electrically accessible even down to the single-defect level [116]. When combined with first-principles calculations, photocurrent spectra may further help narrow down candidate microscopic defect models [117]. Such characterization may help assess whether these photocurrent-active defects should be suppressed as background-current sources or explored as potential candidates for electrically detected sensing. Furthermore, wavelength-dependent ionization processes can provide photoelectrical detection selectivity. For spin-active defects, this wavelength selectivity can be useful for improving the selectivity of magnetic-resonance signals toward target defects, even when spectral filtering of photoluminescence is ineffective in ODMR [116,118,119]. Further, PDMR can provide detection selectivity through the wavelength dependence of ionization processes [118], which can be useful for improving signal contrast even when spectral filtering of the photoluminescence is ineffective. Overall, these results demonstrate the potential for highly sensitive spin detection, as well as its applicability to a wider variety of defect types. The potential for highly sensitive spin detection, as well as its applicability to a wider variety of defect types, is demonstrated. This highlights the prospects of PDMR in SiC as a compelling avenue for future investigation and development in spin-based quantum technologies.

### 4.2. Doping & carrier transport

For photoelectrical spin detection, the properties of the host semiconductor directly determine the achievable signal-to-noise ratio and therefore the ultimate readout fidelity. Ideally, the material should exhibit (i) low dark current in the absence of illumination, (ii) a low background free-carrier density, and (iii) efficient carrier collection. A low dark current sets the electrical noise floor of the measurement, while a low equilibrium carrier density suppresses leakage pathways that reduce photocurrent contrast. Wide-bandgap semiconductors such as diamond and SiC are therefore particularly suitable for PDMR. Their large bandgaps strongly suppress thermally excited carriers, which minimizes leakage current even at room temperature. In addition, both materials possess indirect bandgaps that suppress radiative recombination and lead to comparatively long carrier lifetimes. These long lifetimes enhance charge-collection efficiency and constitute a key intrinsic materials advantage for photoelectrical spin readout. To fully exploit these intrinsic benefits, however, careful control of material doping is required. Heavy n-type or p-type doping introduces shallow donors or acceptors that supply free carriers even without optical excitation. These carriers increase the dark current and reduce the relative change in photocurrent induced by spin-dependent photoionization, thereby degrading the readout contrast [106,113]. Consequently, high-purity or lightly doped materials are generally favored. When doping is employed, it is primarily used to tune the Fermi level and stabilize the desired charge state of the color center rather than to increase conductivity, since excessive conductivity is detrimental to sensitive photocurrent measurements. In practice, this ideal behavior can further be compromised by recombination centers, extended defects, and trap states that act as carrier sinks and introduce additional fluctuations. Such defect-assisted trapping and charge-exchange processes reduce the collected current and increase low-frequency noise, thereby degrading PDMR contrast and sensitivity, as directly demonstrated in photoelectric NV readout experiments [14]. Recent studies by Todenhagen et al. [120] and Le et al. [121] further show that carrier transport and device geometry strongly influence the overall detection efficiency.

### 4.3. Contact engineering

Forming low-resistance Ohmic contacts on wide-bandgap semiconductors such as diamond and SiC is generally challenging because their large bandgaps and low intrinsic carrier densities hinder efficient carrier injection. Achieving Ohmic behavior typically requires specific metallization schemes and post-deposition treatments, for example Au/Pt/Ti multilayers combined with high-temperature annealing and careful surface preparation to reduce interface barriers [122,123]. For PDMR measurements, however, minimizing contact resistance is often less critical than minimizing dark current, as the dark current directly determines the electrical noise floor and limits the achievable signal-to-noise ratio. Consequently, photoelectrical spin readout does not necessarily require fully Ohmic contacts. Instead, Schottky-type contacts can be advantageous because the Schottky barrier suppresses thermionic leakage and reduces background current, thereby improving photocurrent contrast. In the low-current regime relevant to PDMR, carrier extraction rather than carrier injection governs the detection efficiency. Photogenerated carriers can therefore be efficiently collected by drift in the depletion region formed by back-to-back Schottky junctions when biased above the flat-band condition, enabling high charge-collection efficiency even without strictly Ohmic behavior [60,121].

Practical electrode engineering must also consider material-specific constraints. Nickel is a material of choice for Ohmic and Schottky contacts in 4H-SiC [124], but its magnetization can cause inhomogeneous broadening of the PDMR linewidth [111]. The utilization of non-magnetic materials, such as titanium, has been demonstrated to result in the attainment of narrower linewidths [112]. Leakage current represents another key difference between host materials. In high-purity diamond, the extremely high resistivity ($∼10^{15}\;\Omega \cdot$cm) allows Ohmic contacts to be used with minimal background current [106,110]. In contrast, SiC has a narrower bandgap and shallower impurities, limiting its resistivity to 1–10${\;}^{4}$ Ω⋅cm even in high-purity 4 H-SiC, making leakage current more problematic, especially for single-spin detection [124]. In recent demonstrations, leakage currents have been suppressed to the femtoampere level using back-to-back gold Schottky diodes (Figure 5(**d**)), which provide high barrier heights for both electrons and holes. Furthermore, Schottky contacts have been demonstrated to facilitate charge-collection efficiencies approaching unity under appropriate bias conditions [60,125,126], when the carrier transit time is sufficiently shorter than the trapping or recombination time constants. Indeed, near 100 % charge-collection efficiency has been reported in semiconductor radiation detectors of SiC devices [127–129] and a single-crystal CVD diamond detector [130] and holes in a CVD diamond detector [131]. In the SiC PDMR device reported in Ref [60], with an estimated carrier lifetime of about 1 μs and a carrier transit time on the order of nanoseconds, photocurrent saturation above the designed flat-band condition was reported, supporting the assumption of near-unity charge-collection efficiency in the device.

The ability to operate efficiently without strictly Ohmic contacts offers additional advantages for defect and device engineering. Forming Ohmic contacts in SiC typically requires high-dose ion implantation and high-temperature annealing [124], processes that can introduce damage and degrade spin coherence. Schottky-based architectures therefore provide a practical and low-damage alternative for photoelectrically detected spin readout, offering both reduced leakage and improved compatibility with sensitive quantum defects.

### 4.4. Charge state stability

Photoelectrical readout in PDMR is generally based on spin-dependent photoionization processes that involve charge-state conversion of the defect under optical illumination. The generated photocurrent originates from these photoinduced capture and emission events. Therefore, controlled charge-state conversion dynamics are essential for producing a photoelectrical signal [120]. This requirement differs from ODMR, where a stable defect charge state is typically desired because ionization reduces fluorescence contrast and degrades the readout fidelity [12,69]. In PDMR, by contrast, the charge-state transition itself forms the signal mechanism. Nevertheless, complete charge instability is also detrimental, since excessive ionization suppresses the available spin-active population and reduces the photocurrent contrast. Consequently, both modalities require an optimal balance between charge stability and controllable conversion dynamics. Although the readout process occurs under nonequilibrium optical excitation, the equilibrium charge-state distribution is influenced by the Fermi level position, which is governed by impurity doping and surface termination. The Fermi level determines the initial population of spin-active defects and the availability of carriers, thereby affecting both fluorescence and photocurrent generation. These considerations apply broadly to different material platforms, including NV center in diamond and V${\;}_{\mathrm{Si}}$ in SiC, although the optimal doping and surface conditions depend on the specific defect levels in each host. Thus, charge-state engineering is a central design parameter for both diamond- and SiC-based quantum sensors, albeit with different optimization criteria for optical and photoelectrical detection.

## 5. Quantum scanning probe implementations

### 5.1. Tip fabrication

The fabrication of monolithic diamond scanning probes typically relies on plasma-based etching processes, such as reactive ion etching (RIE) or inductively coupled plasma (ICP) etching, to define high-aspect-ratio nanopillars and sharp tips [132–135]. While these techniques enable precise nanostructuring, plasma exposure can introduce sub-surface damage, including vacancy complexes, amorphized layers, surface graphitization, and charge traps near the diamond surface [136–139]. Such defects are particularly detrimental for shallow NV centers, whose spin properties are strongly influenced by the near-surface environment [140]. Experimental studies have demonstrated that oxygen plasma treatment can substantially modify the structural and electronic environment of shallow NV centers [141]. In particular, plasma exposure has been reported to cause the disappearance or modification of pre-existing shallow NVs, indicating significant alteration of the near-surface defect landscape [137]. Such observations are consistent with plasma-induced displacement damage and restructuring of vacancy-related complexes in the sub-surface region. Oxygen plasma processing can introduce or activate electrically active defects and charge traps near the diamond surface [137,138]. Because shallow NV centers reside within the region most strongly affected by surface modification, ion-induced defect formation can perturb their local electrostatic and magnetic environment. Near-surface defect states and charge traps can modify the local band bending and influence the occupation of the NV charge-transition level (NV${\;}^{0/-}$), thereby affecting the stability of the NV${\;}^{-}$ charge state [140,141]. Moreover, increased densities of surface and sub-surface defects are known to contribute to electric-field noise and magnetic noise that couple to shallow NV spins [142–144]. Because the strength of surface-induced decoherence scales strongly with NV depth, plasma-induced near-surface damage can reduce spin coherence times ($T_{2}$), broaden ODMR linewidths, and degrade magnetic sensitivity [143,145,146]. Importantly, the extent of these effects depends strongly on plasma parameters, and carefully optimized low-damage ICP processes have been shown to mitigate such degradation [138]. Therefore, from a materials engineering perspective, controlling plasma-induced structural and electronic modifications of the near-surface region is essential for achieving reproducible shallow NV coherence. Optimization requires balancing nanostructuring fidelity with minimization of displacement damage, defect complex formation, and charge-trap density in the surface regime.

### 5.2. Surface termination

Shallow NV are essential for nanoscale magnetic imaging in scanning NV microscopy where spatial resolution scales with the sensor-sample distance. However, at such shallow depths, NV properties become strongly influenced by surface states. Surface termination plays a critical role in determining charge stability, spin coherence, and electric-field noise, thereby directly impacting magnetic sensitivity and measurement reliability. The charge state of the NV is governed by the local band structure near the diamond surface [140,147]. Surface termination modifies the electron affinity (EA) and induces band bending through surface dipoles and charge transfer, thereby shifting the Fermi level relative to the NV charge-transition level (NV${\;}^{0/-}$). These effects can stabilize or destabilize NV${\;}^{-}$ state required for quantum sensing [2]. Oxygen termination typically yields a positive electron affinity (PEA) and moderate downward band bending at the surface [148]. In this configuration, the near-surface conduction band edge is lowered relative to vacuum, and the Fermi level remains sufficiently high within the band gap to favor occupation of the NV${\;}^{-}$ state. As a result, oxygen-terminated diamond generally supports stable NV${\;}^{-}$ under ambient conditions and has become the standard surface preparation for shallow-NV experiments [141]. In contrast, hydrogen termination produces negative electron affinity (NEA) and typically induces upward band bending, often accompanied by a surface transfer-doping mechanism that forms a two-dimensional hole accumulation layer (2DHG) [147]. The upward band bending shifts the near-surface Fermi level toward the valence band maximum, reducing electron availability at shallow depths. Consequently, shallow NV${\;}^{-}$ can become unstable, favoring conversion to NV${\;}^{0}$, which leads to reduced ODMR contrast and charge-state instability [149]. Fluorine termination introduces strong surface dipoles due to the high electronegativity of fluorine, resulting in enhanced positive electron affinity and can induce strong downward band bending depending on surface chemistry [150,151]. Importantly, band bending not only determines the equilibrium charge state but also modulates the occupation of surface trap states and fluctuating charge centers. The resulting noise sources scale strongly with NV depth and become dominant for shallow NV centers [142,144]. Therefore, surface termination engineering is central to controlling both NV${\;}^{-}$ charge stability and noise suppression in shallow NV-based magnetometry.

### 5.3. Limitations of quantum scanning probe

Scanning NV probe microscopy has enabled quantitative imaging of magnetic and electric fields with nanoscale spatial resolution under ambient conditions and has become one of the most powerful solid-state quantum sensing platforms. In most current implementations, a single NV center located near the apex of a diamond tip is optically initialized and read out using confocal microscopy, while microwave fields are applied for coherent spin control. This configuration has demonstrated high sensitivity and has been widely used for nanoscale magnetometry and current imaging. Nevertheless, several practical constraints arise in scanning-probe implementations. First, ODMR requires both optical excitation for spin initialization and microwave irradiation for spin manipulation. More importantly, the readout signal relies on fluorescence detection. Because the sensing signal is carried by photons, efficient detection requires high-numerical-aperture objectives, optical filters, and photon-counting detectors. These free-space optical components typically dominate the system size and complexity, making miniaturization or on-chip integration challenging. In addition, the high refractive indices of diamond and SiC lead to strong total internal reflection, which fundamentally limits photon extraction efficiency even with optimized optical designs. As a result, only a small fraction of the emitted photons contributes to the detected signal. These photon-collection constraints complicate probe integration and scalability. Consequently, while ODMR-based scanning probes are mature and highly effective, their scalability and device integration are often limited by practical optical considerations rather than by the intrinsic spin physics alone.

Photoelectrical readout based on PDMR replaces fluorescence collection with current detection. Although optical excitation and microwave control are still required, the sensing signal is carried by charge and can be extracted directly through electrodes and processed using integrated electronics. Consequently, bulky photon-collection optics are no longer required, enabling more compact device architectures and improved compatibility with microfabricated probes [152,153]. In contrast, PDMR performance is instead limited by carrier transport properties and electronic noise, highlighting a different set of engineering trade-offs. Therefore, ODMR and PDMR should be regarded as complementary techniques rather than mutually exclusive alternatives. ODMR offers mature, often shot-noise-limited optical detection, whereas PDMR provides advantages in electrical integration and potential miniaturization. These complementary characteristics are particularly relevant for next-generation scanning quantum probes, where compactness and device-level integration are critical. These limitations motivate the exploration of alternative readout strategies that reduce the dependence on fluorescence collection, such as photoelectrical detection based on PDMR.

The single-defect PDMR may in principle be combined with a scanning-probe sensing technique; however, such an implementation remains an open technical challenge. Scanning-probe ODMR sensors based on near-surface NV centers in diamond nanopillars have been widely developed [132,133], and the preservation of spin properties after optimized nanofabrication has been demonstrated [154]. In addition, photocurrent detection using metal/diamond electrode structures (e.g. Ti/Au contacts) has been reported in planar PDMR devices [106,110,152]. These results indicate that the individual technological components required for scanning PDMR probes have been established. Nevertheless, their simultaneous integration into a scanning-probe geometry has not yet been achieved. Nanopillar fabrication processes such as etching, focused ion beam processing, or nanostructuring can introduce crystal damage and near-surface defects, leading to reduced spin coherence and charge-state instability [140]. Furthermore, implementing nanoscale electrodes on submicron-scale pillars is nontrivial. Metallic electrodes placed within tens to hundreds of nanometers of the spin defect may affect the local electrostatic environment, induce band bending, increase background current, and enhance spin relaxation through magnetic Johnson noise [142]. These effects are particularly relevant for pillars with diameters on the order of ∼ 200 nm, where the defect – metal distance can be ∼ 100 nm or less. While nanoscale metallization of sharp probe structures has been demonstrated in other systems, such as SQUID-on-tip devices [155,156], these approaches involve different materials and do not require preservation of spin-defect properties. Therefore, they provide only a limited structural reference and do not directly establish the feasibility of scanning PDMR probes. Taken together, although the key elements of scanning PDMR probes have been demonstrated individually, their integration remains a significant technical challenge. Thus, scanning-probe PDMR should be regarded as a future research direction requiring further advances in nanofabrication, surface engineering, and electrode integration.

### 5.4. PDMR & scanning probe

A key consideration for integrating spin-defect sensors into scanning-probe and device architectures is the local electromagnetic and material environment in which the sensor operates. In many nanoscale measurement scenarios, such as probing local current distributions or electronic transport, the sensing element must be positioned in close proximity to metallic electrodes, conductive layers, or nanostructured devices. In such environments, fluorescence-based ODMR readout can be significantly affected by optical quenching mechanisms, including nonradiative decay channels and plasmonic interactions near metal surfaces [133,157–159]. These effects can reduce the detectable fluorescence signal and degrade readout reliability, particularly for near-surface defects or scanning-probe configurations, although plasmonic enhancement is typically restricted to carefully engineered configurations [160] and is not generally applicable to practical sensing environments. In contrast, PDMR employs spin-dependent photoionization processes in which optical excitation generates charge carriers that are detected electrically as a photocurrent. Because the readout signal is electrical rather than optical, PDMR is less directly dependent on efficient fluorescence collection and photon-collection limitations. Although optical excitation remains necessary, electrical readout may provide an alternative pathway for accessing spin information through electrically contacted device structures. Such an approach may be particularly relevant in extreme near-field sensing geometries where conductive materials are intentionally placed in close proximity to spin defects and fluorescence-based readout becomes increasingly challenging due to quenching effects. Importantly, this does not imply that PDMR universally outperforms ODMR. Rather, PDMR may offer distinct advantages in specific scenarios, particularly in electrically integrated architectures and scanning-probe geometries where optical readout is compromised. Nevertheless, the relative advantages and limitations of ODMR and PDMR in such environments remain an active topic of investigation, and the superiority of either approach has not yet been established. Realizing scanning-probe PDMR devices remains technically challenging. While individual components – such as ODMR-based scanning probes [132,133], photocurrent detection in planar PDMR devices [14], and nanoscale metallization techniques – have been demonstrated, their integration into a single platform is still an open problem. Fabrication processes can introduce surface damage and defects that degrade spin coherence and charge stability [140], and the presence of nearby metallic electrodes may influence the local electrostatic environment and induce additional noise sources [142]. In addition, conductive structures may modify the local electrostatic landscape through surface band bending, charge trapping, and Fermi-level shifts, potentially affecting defect charge-state stability and spin-dependent photoionization processes. Increased background photocurrent, leakage current, and low-frequency electrical noise may further reduce the achievable spin-readout contrast and sensitivity. We therefore emphasize that scanning-probe PDMR should be regarded as a promising but technically demanding direction, rather than a fully established technique. Future progress will require coordinated advances in defect engineering, electrode design, and noise mitigation strategies.

## 6. Comparison of optical and photoelectrical readout

### 6.1. Technical features and device requirements

Optical readout of solid-state spin defects is most commonly implemented using a confocal microscope configuration, which also forms the basis of scanning-probe NV magnetometry. In confocal detection, a tightly focused laser excites a diffraction-limited volume and the emitted fluorescence is collected through the same objective lens and detected using single-photon detectors such as avalanche photodiodes (APDs). Spatial filtering with a pinhole suppresses out-of-focus background light, enabling high signal contrast and near shot-noise-limited photon counting [161]. This configuration provides several key advantages that are particularly important for scanning-probe sensing: high spatial resolution, efficient background rejection, and compatibility with single-defect measurements at the tip apex. Consequently, confocal detection has become the standard platform for single-NV magnetometry and nanoscale imaging, where precise optical addressing and low technical noise are essential. For ensemble sensing, wide-field approaches are often adopted to increase the detected photon rate. These may use camera-based imaging for parallel, spatially resolved readout or integrating photodiodes that collect the total fluorescence intensity [74,104,162,163]. In the latter case, the analog signal is amplified using a transimpedance amplifier and frequently processed using differential or lock-in detection to suppress laser intensity noise. Although these electronics introduce additional technical noise, the dominant limitation typically remains photon shot noise, and the sensitivity scales favorably with the number of emitters. Electrical detection provides an alternative approach in which spin-dependent processes are converted into measurable electrical signals. Such techniques include EDMR and PDMR. In PDMR, optical excitation is used to generate carriers through photoionization while the resulting signal is detected electrically through device electrodes (Figure 1(**c**)), effectively replacing photon counting with photocurrent measurement. Because the signal is an photoelectrical current rather than discrete photon events, low-noise amplification electronics are inherently required. The photocurrent is commonly amplified using a transimpedance amplifier and detected using lock-in [106,110], DC [109], or digitizer-based schemes [113] depending on the protocol. In addition to intrinsic carrier shot noise, the measurement can be affected by amplifier noise, contact resistance fluctuations, carrier recombination losses, and low-frequency transport (1/f) noise. These device- and electronics-dependent contributions often determine the practical noise floor and can exceed the fundamental shot noise. From an integration perspective, the two modalities also differ substantially. Optical detection typically relies on optical components such as objective lenses, filters, and photon detectors. While parts of the optical path can be fiber-coupled or integrated using compact photonic components, optical addressing of the defect generally requires optical access near the sensing region, which can contribute to system-level integration constraints depending on the device geometry. In contrast, PDMR detects spin-dependent photoionization as a photocurrent at the device electrodes, which can, in principle, be interfaced with on-chip electrodes and compact electronics [153], although practical implementation requires careful device engineering and low-noise electronic design. This compatibility with semiconductor processing suggests potential for more compact or integrated sensor architectures, although significant technical challenges remain. Nevertheless, it is important to emphasize that PDMR still requires optical excitation for spin initialization and photoionization, and therefore does not eliminate the need for optical access, but rather replaces only the fluorescence detection pathway. Overall, confocal ODMR provides robust, near shot-noise-limited performance and remains the dominant technique for nanoscale and scanning-probe sensing, whereas PDMR-based photocurrent detection offers potential advantages in device-level signal collection and integration, but its performance remains sensitive to materials quality and electronic noise. Continued advances in materials quality, contacts, and low-noise electronics are therefore essential to fully exploit the potential of PDMR-based spin readout.

### 6.2. Readout fidelity

In the quantum sensing community, spin-readout performance is commonly characterized using the readout-noise factor, $\sigma_{R}$, introduced by Barry *et al* [2]. In this work, we refer to its inverse of read-out factor of $\sigma_{R}^{-1}$ as the readout fidelity. This quantity characterizes the excess noise associated with spin-state readout relative to the spin-projection-noise limit and is distinct from quantum-state-overlap fidelity. Since the readout-noise factor is satisfied the condition of $\sigma_{R}\geq 1$, the readout fidelity satisfies $0<\sigma_{R}^{-1}\leq 1$. To evaluate the same readout metric in the presence of arbitrary experimental noise, we consider two spin-dependent mean signals, $S_{0}$ and $S_{1}$, with corresponding conditional variances $\sigma_{0}^{2}$ and $\sigma_{1}^{2}$. At the maximum-slope operating point, the inverse readout-noise factor is expressed as

(18) $$\sigma_{R}^{-1}={\left[1+\frac{2(\sigma_{0}^{2}+\sigma_{1}^{2})}{{\left(S_{0}-S_{1}\right)}^{2}}\right]}^{-1/2}.$$

This generalized expression permits $\sigma_{R}^{-1}$ to be evaluated using experimentally measured noise, including technical and electronic noise. By contrast, when the detection is assumed to be limited under shot-noise-limited detection conditions, $\sigma_{i}^{2}=S_{i}$. Under this shot-noise-limited assumption, $\sigma_{R}^{-1}$ reduces to

(19) $$\sigma_{R}^{-1}={\left[1+\frac{1}{C^{2}\overset{ˉ}{S}}\right]}^{-1/2},$$

where $C=\frac{|S_{0}-S_{1}|}{S_{0}+S_{1}}$ is the symmetric readout contrast and $\overset{ˉ}{S}=\frac{S_{0}+S_{1}}{2}$ is the mean number of detected photons or carriers per readout cycle. In the low-readout-fidelity limit, $C^{2}\overset{ˉ}{S}\ll 1$, this expression becomes $\sigma_{R}^{-1}≃C\sqrt{\overset{ˉ}{S}}.$ When a detection rate R is reported, the detected number per readout cycle is obtained as $\overset{ˉ}{S}=Rt_{\mathrm{ro}}$, where $t_{\mathrm{ro}}$ is the readout duration. Based on this common definition, Table 3 summarizes values of $\sigma_{R}^{-1}$ for optical and photoelectrical readout of NV centers in diamond and V${\;}_{\mathrm{Si}}$ centers in SiC. The tabulated values were obtained by taking the reciprocal of a reported $\sigma_{R}$, recalculating $\sigma_{R}^{-1}$ from reported contrast and detection parameters, or converting a reported or experimentally measured SNR to a per-readout-cycle quantity using the generalized expression above. The evaluation procedure used for each entry is explicitly indicated in the table. It should be noted that Table 3 is intended to provide a quantitative comparison of reported readout metrics under a unified definition and does not imply that PDMR currently outperforms ODMR. Table 3 shows that the reported inverse readout-noise factors vary substantially among both single-defect and ensemble measurements. The ensemble values should not be interpreted as exhibiting a universal $\sqrt{N}$ enhancement of $\sigma_{R}^{-1}$. In the sensitivity formalism, the number of sensing spins, N, and the readout-noise factor, $\sigma_{R}$, represent distinct contributions. Although the total measurement SNR may improve approximately as $\sqrt{N}$, the per-readout-cycle fidelity also depends on the collection or carrier-detection efficiency, readout contrast, background signal, readout duration, and technical noise. For optical detection, many of the tabulated values were obtained under a photon-shot-noise-limited assumption. Practical ODMR measurements may nevertheless also be affected by laser-intensity fluctuations, microwave instability, detector noise, and background fluorescence. Similarly, practical PDMR measurements can be influenced by carrier-transport and recombination processes, contact resistance, interface instabilities, transimpedance-amplifier noise, lock-in-amplifier noise, and low-frequency electrical fluctuations. The spread of the reported values therefore reflects not only differences between optical and electrical detection, but also differences in readout protocols, measurement durations, device structures, experimental conditions, and estimation procedures. These considerations are particularly important for PDMR because its readout performance depends strongly on materials engineering, carrier transport, electrical-contact design, and readout electronics. These considerations are particularly important for PDMR because its readout performance depends strongly on materials engineering, carrier transport, electrical-contact design, and readout electronics. Theoretical analyses have suggested that photoelectrical readout may achieve competitive magnetic-field sensitivity under optimized noise and device conditions [109]. However, current experimental implementations remain affected by excess noise associated with carrier transport, interfaces and contacts, and readout electronics [107,109,170]. Further device and electronic optimization is therefore required to establish whether the predicted sensitivity advantages can be realized experimentally. Accordingly, Table 3 should be interpreted as a summary of protocol-specific reported readout metrics rather than as a direct ranking of ODMR and PDMR or as evidence that photoelectrical readout presently provides superior overall sensing performance.

**Table 3.** Comparison of optical and electrical detection methods of NV centers in diamond and V${\;}_{\mathrm{Si}}$ in SiC. Readout-fidelity values ($\sigma_{R}^{-1}$) are presented following the formalism of Barry *et al*. [2]. The column ‘evaluation basis’ indicates whether each value was obtained from reported $\sigma_{R}^{-1}$ or derived from experimentally measured SNRs ($\sigma_{REx}^{-1}$).

Material	Detection Method	Readout Protocol	Single/Ensemble	$\sigma_{R}^{-1}$	$\sigma_{REx}^{-1}$	Evaluation Basis	Reference
NV center	Optical detection	ODMR	Single	9.4 $\times 10^{-2}$		Reported $\sigma_{R}$ = 10.6	[164]
NV center	Optical detection	Spin-to-charge conversion	Single	3.6 $\times 10^{-1}$		Reported $\sigma_{R}$ = 2.76	[164]
NV center	Optical detection	ODMR	Single	2.9 $\times 10^{-2}$		Reported $\sigma_{R}$ = 35	[165]
NV center	Optical detection	ODMR	Single	1.3 $\times 10^{-2}$		Reported $\sigma_{R}$ = 80	[166]
NV center	Optical detection	ODMR	Single	2.1 $\times 10^{-2}$		Reported $\sigma_{R}$ = 48	[167]
NV center	Optical detection	ODMR	Single	1.9 $\times 10^{-2}$		Reported $\sigma_{R}$ = 54	[168]
NV center	Optical detection	Ancilla-assisted repetitive readout	Single	9.1 $\times 10^{-1}$		Reported $\sigma_{R}$ = 1.1	[169]
NV center	Optical detection	ODMR	Ensemble	1 $\times 10^{-3}$		Reported $\sigma_{R}∼$ 1000	[163]
NV center	Photoelectrical detection	PDMR	Ensemble	1.3 $\times 10^{-4}$		Contrast + detection rate	[106]
NV center	Photoelectrical detection	PDMR	Ensemble	2 $\times 10^{-2}$		Contrast + detection rate	[107]
NV center	Optical detection	ODMR	Single		1.6 $\times 10^{-3}$	Measured SNR	[152]
NV center	Photoelectrical detection	PDMR	Single		1.5 $\times 10^{-3}$	Measured SNR	[152]
V${\;}_{\mathrm{Si}}$	Optical detection	ODMR	Single		8.0 $\times 10^{-4}$	Measured SNR	[60]
V${\;}_{\mathrm{Si}}$	Photoelectrical detection	PDMR	Single		1.3 $\times 10^{-3}$	Measured SNR	[60]

### 6.3. Sensitivity

A fair comparison of magnetic-field sensitivity between PDMR and ODMR is inherently complex. As discussed in sections 6.1 and 6.2, ODMR measurements frequently approach the photon shot-noise limit, which originates from the Poisson statistics of detected photons, whereas PDMR relies on electrical detection of photocurrent and its sensitivity in practical experiments is often constrained by additional noise sources arising from carrier transport within the host diamond and from the detection electronics. These noise sources frequently dominate over the intrinsic current shot noise and therefore prevent the measurement from reaching the current shot-noise limit. Consequently, the two modalities are not always directly comparable under identical experimental conditions. Nevertheless, several recent studies have performed direct side-by-side comparisons using identical excitation conditions and the same diamond samples while varying only the detection method. Zheng *et* *al.* [115] demonstrated microwave-free magnetometry at the ground-state level anticrossing (∼ 102.4 mT). They reported that the noise floor of PDMR was approximately four times higher than that of ODMR. However, the sensitivity normalized to the excitation volume was reported to be higher for PDMR, which is attributable to its more localized effective detection volume [152,170]. In fluorescence-based confocal ODMR, the effective detection volume is determined not only by the excitation profile but also by the fluorescence collection process, including the finite pinhole size and the emission point-spread function. For color centers such as NV and V${\;}_{\mathrm{Si}}$, the Stokes shift further broadens the effective detection volume because the fluorescence wavelength is longer than the excitation wavelength. In contrast, PDMR directly detects photocurrent generated at the excitation site and is therefore not affected by fluorescence collection broadening. Consequently, PDMR approaches the spatial resolution expected for an ideal nonlinear optical microscopy technique and can provide a smaller effective sensing volume than confocal ODMR under comparable excitation conditions. Recent work by one of the authors directly compared the spatial resolution of photoelectric and confocal optical readout using a single NV center and reported a narrower image full width at half maximum for photoelectric detection under identical excitation conditions, supporting this interpretation [152]. This quantity should not, however, be interpreted as evidence that PDMR intrinsically generates a larger magnetic-field signal from an identical sensing volume. Rather, it reflects the ability to achieve comparable sensing performance while probing a smaller effective sensing volume. Similarly, Hruby *et* *al.* [170] performed simultaneous continuous-wave measurements using both PDMR and ODMR on the same sample. By employing a yellow-green laser (561 nm) for PDMR to suppress background photoexcitation from other defects such as P1 centers, they reported that, under certain experimental conditions, photoelectric readout can achieve a shot-noise-limited magnetic-field sensitivity that is improved compared with ODMR. These results indicate that PDMR-based photoelectrical readout should be regarded as a complementary approach for quantum sensing rather than a universally superior alternative to ODMR. Both ODMR and PDMR require optical excitation; however, PDMR-based photoelectric detection may simplify certain chip-scale implementations by removing the need for fluorescence collection optics, while introducing additional design constraints such as electrode structures and routing. In addition, spatially confined carrier generation may be advantageous in localized probing, spatially selective sensing, or integrated sensing architectures where the effective sensing volume must remain small. In such situations, sensitivity normalized to sensing volume provides a useful complementary figure of merit because absolute magnetic-field sensitivity alone does not allow a fair comparison between sensing platforms operating with substantially different sensing volumes or numbers of addressed color centers.

### 6.4. Scalability and sensitivity enhancement

A primary route to improving sensitivity in solid-state quantum sensors is to increase the number of active color centers participating in the measurement. In ensemble-based quantum sensing using color centers, the detected signal generally scales approximately linearly with the number of emitters N, while the fundamental shot noise scales as $\sqrt{N}$. In ODMR, for example, the detected fluorescence rate follows this scaling behavior. In ODMR, the detected fluorescence rate scales approximately linearly with the number of emitters N, while photon shot noise scales as $\sqrt{N}$. Consequently, the readout efficiency follows the well-known scaling $\sigma_{R}^{-1}\propto C\sqrt{R}\propto \sqrt{N}$, enabling straightforward sensitivity enhancement through ensemble measurements [2,83]. This favorable statistical scaling has motivated the widespread use of wide-field or bulk optical detection, where large sensing volumes containing many defects can be interrogated simultaneously. In practice, however, ensemble scaling in ODMR is not purely statistical. Increasing the defect density can introduce additional dephasing mechanisms such as dipolar spin-spin interactions, strain inhomogeneity, and optical power broadening, which shorten coherence times and reduce contrast [44,171,172]. Therefore, an optimal balance between emitter number and material quality is required to fully benefit from the ideal $\sqrt{N}$ scaling. As discussed in Sec. 3.3, sensitivity enhancement is not limited to increasing the number of emitters. Pulse-sequence engineering can extend the effective $T_{2}$, thereby improving sensitivity through longer phase accumulation. Correlation-based approaches such as Qdyne further enable frequency estimation beyond the conventional $T_{2}$ limit by coherently combining repeated measurements. Moreover, the use of correlated or entangled spin states provides a route beyond the standard quantum limit. Techniques including spin squeezing and entanglement-assisted sensing have recently demonstrated experimentally improved sensitivity or enhanced spatial resolution in solid-state ensembles, approaching scaling beyond the conventional $1/\sqrt{N}$ behavior toward the Heisenberg limit (1/N) [173,174]. These results indicate that sensitivity gains can arise not only from statistical averaging but also from quantum control and many-body correlations.

For PDMR, a similar $\sqrt{N}$ scaling is expected in principle because the photocurrent increases proportionally with the number of photoionization events and thus with the number of defects. In practice, however, the signal is an analog current that must be transported through the host semiconductor before reaching the external detection electronics. During this transport process, carriers may recombine, become trapped at defects, or be lost due to finite carrier lifetime, reducing the collection efficiency and introducing additional fluctuations. Consequently, the achievable signal is strongly influenced by host-material properties such as carrier lifetime, mobility, and trap density. In addition, photoelectrical readout requires amplification using transimpedance amplifiers and other circuitry, which introduces further electronic noise as discussed in Sec. 6.1. As a result, both carrier-transport losses inside the material and electronic noise in the measurement chain can exceed the intrinsic shot noise. These effects often prevent ideal $\sqrt{N}$ scaling and make the achievable sensitivity dependent not only on defect number but also on material quality, device structure, and readout electronics. Therefore, improving PDMR sensitivity relies on both increasing the number of color centers and optimizing the host material and device design, including longer carrier lifetimes, reduced trap densities, efficient carrier extraction, stable contacts, and low-noise amplification electronics. With appropriate materials optimization and device engineering, ensemble PDMR may approach similar statistical scaling behavior. However, the realization of such scaling remains strongly dependent on carrier transport efficiency, defect density control, and low-noise electronics.

### 6.5. Inherent limitations and practical considerations

Although photoelectrical readout replaces photon detection with current measurement, it does not eliminate the need for optical excitation. PDMR still relies on laser illumination to initialize the spin state and to induce spin-dependent photoionization and charge-state conversion. Consequently, optical access remains necessary in both modalities, and detection substitutes only the fluorescence readout pathway rather than the entire optical infrastructure. The two approaches also differ fundamentally in their detection physics. ODMR detects discrete fluorescence photons immediately after emission, and the signal is therefore governed primarily by photon collection efficiency and photon shot noise. In contrast, PDMR measures an analog photocurrent generated by charge carriers that must be transported through the host semiconductor before reaching the electrodes. As a result, the readout becomes sensitive to carrier recombination, trapping, finite carrier lifetime, and background photocurrents, in addition to noise introduced by the amplification electronics. These transport- and device-related processes can reduce collection efficiency and introduce excess fluctuations beyond the intrinsic shot noise. From a practical standpoint, ODMR typically provides stable, near shot-noise-limited performance with mature and well-established optical hardware. Photoelectrical detection, while offering potential advantages in certain device architectures, remains strongly dependent on host-material quality, device engineering, and low-noise electronics. The realization of fully optimized PDMR-based sensors therefore continues to require significant advances in materials control and electronic design.

## 7. Demonstrations of the quantum sensor

### 7.1. Diamond NV quantum sensor

The basis of the magnetometry using scanning NV-center microscopy was reported in 2008 [5,8,83], which enables the characterization of the stray magnetic field with high sensitivity and atomic-size spatial resolution, without any back-reaction on the sample. The technique has been demonstrated to be operational under ambient conditions, as well as at cryogenic temperatures [175]. The merits of this technique are twofold, in comparison to both traditional magnetic force microscopy and magnetic resonance microscopy, and it has been demonstrated to engender significant advances in understanding the various magnetic natures of materials and nanostructures. In 2012, the first documented instance of stray field imaging in magnetic storage media was reported [11,133]. Subsequently, the magnetic imaging of living cells, i.e. magnetotactic bacteria, was achieved using a single electron spin [176]. While Faraday- and Kerr-based magneto-optical techniques can visualize magnetization patterns in thin films, they probe magnetization indirectly through material-dependent optical responses. In contrast, solid-state quantum sensors such as NV centers measure the stray magnetic field itself through Zeeman shifts of the spin resonance frequency, enabling direct and quantitative magnetic field imaging with nanoscale spatial resolution [177]. This distinction is particularly important when quantitative reconstruction of stray-field distributions is required. Scanning NV microscopy is now commercially available, with integrated systems offered by several companies. Nevertheless, its widespread adoption remains limited at the current stage. This is primarily due to the relatively high system cost, the limited robustness and lifetime of NV probes, and the comparatively low imaging throughput inherent to point-by-point scanning and ODMR acquisition. Furthermore, the requirement of microwave excitation, precise optical alignment, and advanced data analysis increases experimental complexity [7,178]. Consequently, although scanning NV microscopy represents a powerful platform for quantitative nanoscale magnetic field imaging, it is presently more suited to specialized investigations than to routine magnetic characterization.

To date, stray-field imaging techniques have been applied to a wide range of magnetic systems, including bulk materials, thin films and patterned structures with ferromagnetic, ferrimagnetic and antiferromagnetic metals, oxides and 2D materials. As demonstrated in seminal studies, the imaging of vortex cores in patterned Ni-Fe alloys with in-plane magnetization [179] and domain walls in perpendicularly magnetized Ta/CoFeB/MgO films [180] was a significant milestone in the field. The technique has also been employed to reveal the internal structures of the domain walls in insulating ferrimagnetic Tm${\;}_{3}$Fe${\;}_{5}$ O${\;}_{12}$ films [181] and is now widely used to characterize stray field patterns in devices [182,183]. In non-collinear magnets, the local dynamics of topological defects were mapped in bulk FeGe [184] and stray fields were reported for BiFeO${\;}_{3}$ films [185]. The existing body of research on collinear antiferromagnets encompasses the following studies: first, the domain wall imaging of synthetic and bulk Cr${\;}_{2}$O${\;}_{3}$ [186] and second, the investigation of topological spin textures in Fe${\;}_{2}$O${\;}_{3}$ [187]. The technique has recently become popular for imaging stray fields in patterned antiferromagnetic structures [188–190] and in 2D van der Waals (vdW) magnets [191]. The technique has been demonstrated to be a highly effective state-of-the-art magnetometry for the investigation of emergent magnetism in moiré magnetic superlattices [192,193].

### 7.2. SiC quantum sensor

For V${\;}_{\mathrm{Si}}$-based sensors, the measurable physical quantities demonstrated to date using ODMR-based techniques include magnetic field, temperature, and electric field. Reported sensitivities include an ensemble DC sensitivity of 4 nT/$\sqrt{\mathrm{Hz}}$ [62], a single-defect AC sensitivity of 358 μT/$\sqrt{\mathrm{Hz}}$ [64], and an ensemble AC sensitivity of 1.4 μT/$\sqrt{\mathrm{Hz}}$ [194]. Proof-of-principle stray-field measurements of magnons using V2 defects [195] have been reported; however, systematic sensitivity benchmarking for these implementations has not yet been fully established. Experimental demonstrations further support the feasibility of direct sensing within SiC device structures. This is achieved by V${\;}_{\mathrm{Si}}$ quantum sensors formed at specific locations within the device after device fabrication process. Since V${\;}_{\mathrm{Si}}$ formation will degrade device performance, minimizing device degradation is critical for accurate sensing. Therefore, there is a need for V${\;}_{\mathrm{Si}}$ selective formation techniques such as particle beam writing [196,197] or femtosecond laser irradiation [198], and for controlling the amount of V${\;}_{\mathrm{Si}}$ to a minimum although this reduces sensor sensitivity. In this respect, this measurement method is not non-invasive. Furthermore, in power devices, it is necessary to maintain the intended structure to prevent device failure caused by high electric field concentration. For example, it is not possible to introduce a SIL structure to improve photon-collection efficiency. Therefore, improvements of sensor sensitivity achieved through quantum operations become even more important [199]. For ODMR, florescence from spin defects is used for sensing. For this reason, measurements cannot be taken under electrodes, which limits the areas that can be measured. As a device structure becomes more complex, for example, in trench-type or super-junction-type devices, structural factors may further limit the measurable region. On the other hand, PDMR may enable sensing under the electrode if quantum sensors can be selectively formed in such regions, which is achievable by adjusting the ion energy. There are no physical constraints on excitation methods as strict as those on detection methods. It is possible to choose a method where excitation light is incident from the side of the device, as was demonstrated with NV center-based quantum sensor [200]. This eliminates the difficulty of optically exciting defects located directly beneath the electrode. However, since PDMR detects current as a signal, it becomes difficult to identify the origin of the signal if there are other current sources (noise) contributing to the detected signal. To improve the spatial resolution in PDMR, it is necessary to reduce noise sources as much as possible and separate noise from the signal. Despite these limitations, the direct sensing techniques using ODMR and PDMR can still be usable because it covers some of the areas that are critical for power devices.

Temperature monitoring has been achieved in a planar-type SiC pn diode, where the temperature rise induced by current flow was detected using V${\;}_{\mathrm{Si}}$ defects via an ODMR-based technique [201]. More recently, electric-field detection has been demonstrated in a vertical pn diode configuration, also based on ODMR readout [202]. These results indicate that both thermal and electric field quantities can be accessed within functioning SiC device structures, providing experimental evidence supporting the feasibility of in-device sensing using V${\;}_{\mathrm{Si}}$ defects. In these papers, ODMR was used. Overall, many SiC-based sensing demonstrations remain at the proof-of-principle stage, and systematic quantitative performance optimization is an important direction for future work.

Beyond sensing performance, the materials platform itself offers distinctive advantages. As shown in Table 2, 8-inch SiC wafers are commercially available, and research scale demonstrations have extended wafer diameters to 12 inches. Furthermore, we can utilize a matured device fabrication technique. In electrically detected sensing methods such as PDMR and EDMR, these technologies can be directly leveraged. Specifically, lithography enables patterned electrode formation, metal deposition is used for electrical contacts, doping and epitaxial growth allow carrier transport control, and thermal processing is used to form low-resistance ohmic contacts. These processes are essential for controlling device characteristics and play a key role in determining sensor performance and reproducibility. In contrast, ODMR-based sensing relies on optical excitation and fluorescence detection and therefore does not require semiconductor device fabrication processes such as electrode formation or junction engineering. Its implementation is largely independent of device processing, except for material preparation steps such as the defect formation of V${\;}_{\mathrm{Si}}$ [198]. Currently, for ODMR, V${\;}_{\mathrm{Si}}$-based quantum sensors exhibit lower sensitivity compared to NV-based quantum sensors due to intrinsic properties such as lower contrast. However, SiC has advantages in applications that exploit the unique properties of V${\;}_{\mathrm{Si}}$ (for example, their small ZFS enables level anticrossing at low magnetic fields, allowing all-optical magnetic sensing [38], which is being considered for planetary exploration [22]), as well as in sensing methods that require electrical circuits such as EDMR and PDMR, where device fabrication strongly influences sensor performance. From an industrial perspective, compatibility with semiconductor fabrication processes enables integration with electronic circuits, device miniaturization, and wafer-level manufacturing for large-scale production.

## 8. Conclusions and future directions

In this review, we have surveyed solid-state spin defects as versatile quantum sensors and scanning probe with PDMR-based photocurrent readout techniques. We discussed three representative material platforms – NV centers in diamond, V${\;}_{\mathrm{Si}}$ in SiC, and V${\;}_{B}$ centers in hBN – and compared their respective advantages. NV centers in diamond currently provide the most mature and well-balanced performance for room-temperature sensing, while SiC offers wafer-scale fabrication and partial compatibility with conventional semiconductor processing. Meanwhile, hBN represents an emerging two-dimensional platform that enables close proximity between the sensor and target systems, opening opportunities for ultrathin and surface-sensitive quantum probes. Together, these hosts provide a broad materials foundation for scalable solid-state quantum sensing platforms based on ensembles or reproducible defect fabrication. Scanning implementations based on these defects enable local measurements with nanometer-scale spatial resolution and have established quantum probes as powerful tools for condensed-matter physics. Despite these advances, however, the sensitivity of practical scanning probes remains significantly below the intrinsic limits predicted from spin coherence and photon statistics. In particular, commercially available NV-based probes typically operate at sensitivities on the order of μT$/\sqrt{\mathrm{Hz}}$, whereas isolated defects in optimized bulk materials can reach the nT$/\sqrt{\mathrm{Hz}}$ regime. This performance gap originates from two closely connected challenges. First, bringing defects close to surfaces – an essential requirement for high spatial resolution – often degrades spin coherence and charge-state stability due to surface damage, paramagnetic impurities, and electric-field noise. Second, and equally important, conventional fluorescence-based optical readout is often limited by photon-collection efficiency, as only a small fraction of emitted photons can be collected due to limited numerical aperture, optical losses, and refraction at the high-refractive-index interfaces of host materials such as diamond and SiC. Optical engineering approaches such as solid-immersion lenses (SILs), nanopillars, and other nanophotonic structures can significantly improve photon-collection efficiency [203–207]. For example, SIL-based optical structures have demonstrated substantial fluorescence enhancement in defect-based quantum systems [203,206]. However, such approaches typically require nanofabrication in the immediate vicinity of the defect to achieve efficient optical coupling. This can introduce surface damage, strain, and charge noise that may affect the spin and charge properties of near-surface defects, which are particularly critical for sensing applications. In addition, fluorescence-based readout fundamentally relies on photon collection through optical systems, and therefore remains subject to practical constraints such as numerical aperture, optical alignment, and system complexity. In this context, PDMR provides a complementary readout strategy by converting spin-dependent photoionization directly into an electrical signal without relying on photon collection optics. Recent experiments in SiC have demonstrated that photocurrent signals obtained through PDMR can exhibit signal amplification comparable to that achieved using solid-immersion lenses in optical detection schemes [60]. To address this readout bottleneck, we have highlighted PDMR based on spin-dependent photoionization. By converting spin-dependent photoionization processes directly into a photocurrent detected through device electrodes, PDMR provides an alternative readout pathway that reduces the reliance on bulky optical fluorescence collection optics and enables signal readout via electrically contacted device structures. Such configurations include planar semiconductor devices incorporating spin defects within photoconductive or diode-like structures, as well as scanning-probe geometries in which the sensing element is integrated with nanoscale electrodes. In these device architectures, metallic or conductive components are often present in close proximity to the defects, which can significantly suppress fluorescence signals due to optical quenching mechanisms. As a result, fluorescence-based ODMR can be strongly limited in such environments, although plasmonic enhancement is typically restricted to carefully engineered configurations and is not generally applicable to practical sensing environments [160]. In contrast, PDMR enables direct electrical readout that remains operable even in the presence of nearby conductive or metallic structures, where optical readout may become inefficient or impractical. This photocurrent-based detection therefore offers several potential advantages in electrically integrated platforms, including high carrier-collection efficiency under optimized conditions, simplified optical requirements, compact device architectures, and possible integration with scalable semiconductor fabrication [60,109,113,208]

Looking forward, the integration of PDMR-based photocurrent detection schemes with scanning-probe geometries represents a promising direction for next-generation quantum sensors, although practical implementation remains technically challenging. In such architectures, PDMR may offer opportunities for closer local integration of sensing and readout components, potentially supporting compact probe implementations. Realizing this vision will require coordinated advances in several areas, including the creation of high-quality near-surface defects with long coherence times and stable charge states, low-noise electrical amplification, optimized electrode geometries, and microwave delivery schemes compatible with scanning operation. In parallel, improved sensing protocols that balance contrast, bandwidth, and excitation power will be essential for maximizing performance. With continued progress in these areas, scanning probes employing PDMR-based photocurrent detection based on solid-state spin defects may provide a viable pathway toward miniaturized and scalable quantum sensors. In particular, PDMR may offer opportunities for sensing near conductive or metallic interfaces, where fluorescence-based ODMR can be strongly limited by optical quenching at graphene or metal surfaces [133,157–159], although plasmonic enhancement is typically restricted to carefully engineered configurations and is not generally applicable to sensing environments [160]. Such capabilities may broaden opportunities for quantum sensing in condensed-matter systems, conductive heterostructures, and related device platforms.
